# Bulbus Fritillariae Cirrhosae as a Respiratory Medicine: Is There a Potential Drug in the Treatment of COVID-19?

**DOI:** 10.3389/fphar.2021.784335

**Published:** 2022-01-20

**Authors:** Yunyun Quan, Li Li, Zhujun Yin, Shilong Chen, Jing Yi, Jirui Lang, Lu Zhang, Qianhua Yue, Junning Zhao

**Affiliations:** ^1^ Translational Chinese Medicine Key Laboratory of Sichuan Province, Sichuan Academy of Chinese Medicine Sciences, Sichuan Institute for Translational Chinese Medicine, Chengdu, China; ^2^ Department of Pharmacognosy, West China School of Pharmacy Sichuan University, Chengdu, China

**Keywords:** bulbus fritillariae cirrhosae, alkaloids, respiratory diseases, signaling pathways, targets, COVID-19

## Abstract

Bulbus fritillariae cirrhosae (BFC) is one of the most used Chinese medicines for lung disease, and exerts antitussive, expectorant, anti-inflammatory, anti-asthmatic, and antioxidant effects, which is an ideal therapeutic drug for respiratory diseases such as ARDS, COPD, asthma, lung cancer, and pulmonary tuberculosis. Through this review, it is found that the therapeutic mechanism of BFC on respiratory diseases exhibits the characteristics of multi-components, multi-targets, and multi-signaling pathways. In particular, the therapeutic potential of BFC in terms of intervention of “cytokine storm”, STAT, NF-κB, and MAPK signaling pathways, as well as the renin-angiotensin system (RAS) that ACE is involved in. In the “cytokine storm” of SARS-CoV-2 infection there is an intense inflammatory response. ACE2 regulates the RAS by degradation of Ang II produced by ACE, which is associated with SARS-CoV-2. For COVID-19, may it be a potential drug? This review summarized the research progress of BFC in the respiratory diseases, discussed the development potentiality of BFC for the treatment of COVID-19, explained the chemical diversity and biological significance of the alkaloids in BFC, and clarified the material basis, molecular targets, and signaling pathways of BFC for the respiratory diseases. We hope this review can provide insights on the drug discovery of anti-COVID-19.

## Introduction

**FIGURE 1:**
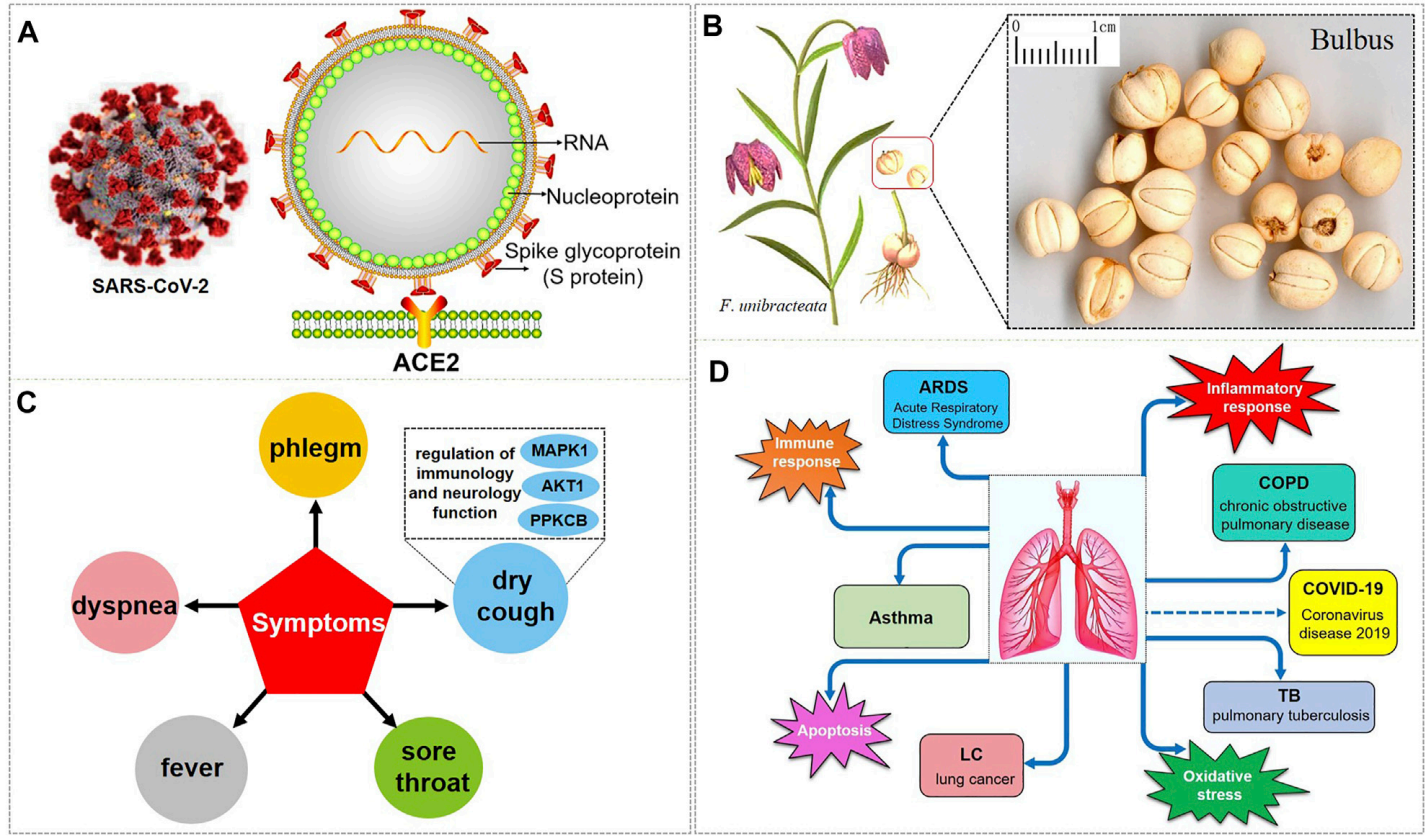
SARS-CoV-2 and the respiratory diseases, biological processes, and symptoms that BFC have an effect on. **(A)** is the 3D and 2D structure of SARS-CoV-2 with its composition. **(B)** is *F. unibracteata*, one of the plant sources of BFC with its bulbus. **(C)** is the symptoms that BFC can relieve. **(D)** is the respiratory diseases and correlative biological processes that can be intervened by BFC.

In December 2019, the infectious disease caused by the novel coronavirus (2019 Novel Coronavirus, 2019-nCoV) began to break out. The epidemic first emerged in Wuhan and quickly swept the world (Holshue et al., 2020; Li et al., 2020a; Zhu et al., 2020; Livingston and Bucher, 2020). On January 30, 2020, the World Health Organization (WHO) declared the epidemic as a “public health emergency of international concern” (Mahase, 2020; Burki, 2020). On February 11, 2020, the International Committee on Taxonomy of Viruses (ICTV) officially designated the virus as “severe acute respiratory syndrome coronavirus 2 (SARS-CoV-2)” (Gorbalenya, 2020). On the same day, the WHO announced that the pneumonia caused by the 2019-nCoV was named “Coronavirus disease 2019, COVID-19” (Jiang et al., 2020; Ghebreyesus, 2019). COVID-19 is an acute respiratory infectious disease caused by SARS-CoV-2 infection, which has become a major threat to the health of people all over the world, and the world is still in the pandemic stage (Wang et al., 2020a; Chavez et al., 2021; Song et al., 2020). Up to now, the number of confirmed COVID-19 cases in the world is 225,187,374, which has exceeded 200 million, and the death toll is 4,640,097, which is nearly 5 million (COVID-19 coronavirus pandemic, 2021; WHO, 2019). SARS-CoV-2, as a single-stranded positive-stranded RNA virus of the β subclass of the coronavirus genus (Wu et al., 2020a; Li et al., 2020b) (Figure 1A), is genetically similar to the 2003 severe acute respiratory syndrome (SARS) coronavirus (SARS-CoV) and the 2012 Middle East respiratory syndrome (MERS) coronavirus (MERS-CoV) (Perlman and Dandekar, 2005; Zumla et al., 2015; Zumla et al., 2016; Dyall et al., 2017; Hui and Zumla, 2019; Patel et al., 2020; Spagnolo et al., 2020). SARS-CoV-2 has about 79% homology with SARS-CoV in sequence, and about 50% with MERS-CoV (Lu et al., 2020a; Zhou et al., 2020a; Gu et al., 2020). SARS-CoV-2 is a virus that is more contagious and can cause acute respiratory distress syndrome (ARDS) discovered after SARS-CoV and MERS-CoV. It mainly causes mild to severe lung infections, and results in acute and highly lethal pneumonia. The clinical symptoms of COVID-19 are similar to those of SARS-CoV and MERS-CoV (Graham et al., 2013; Xu et al., 2020a). The common symptoms of patients with COVID-19 are high fever, dry cough, fatigue, myalgia, sputum production, shortness of breath, and the less common symptoms are sore throat, hemoptysis, chest pain, headache, diarrhea, and vomiting, besides, the most common complications are pneumonia, followed by ARDS, arrhythmia, shock, respiratory failure, and even death from multiple organ failure (Chen et al., 2020a; Huang et al., 2020a; Yang et al., 2020a; Wang et al., 2020b; Wu et al., 2020b; Eastin and Eastin, 2020).

Bulbus fritillariae cirrhosae (BFC), well-known as *ChuanBeimu* or *ChuanBei* in China, is derived from the dried bulbs of six different species of the genus Fritillaria that belong to the family Liliaceae, including *Fritillaria cirrhosa* D. Don (*F. cirrhosa*), *Fritillaria unibracteata* P.K.Hsiao and K.C.Hsia (*F. unibracteata*), *Fritillaria przewalskii* Maxim. ex Batalin (*F. przewalskii*), *Fritillaria delavayi* Franch. (*F. delavayi*), *Fritillaria taipaiensis* P.Y.Li (*F. taipaiensis*), *Fritillaria unibracteata* var. *wabuensis* (S.Y.Tang and S.C.Yueh) Z.D.Liu, Shu Wang, and S.C.Chen (*F. wabuensis*) (Chinese Pharmacopoeia Com, 2015; Chen et al., 2019; Zhang et al., 2016) (Figure 1B). BFC, as a valuable and important traditional Chinese medicine (TCM), has been used as an antitussive, expectorant, and anti-asthmatic drug, and it is considered to be the top-grade one among all Fritillaria species with positive therapeutic effects, low toxicity, and few side effects (Cai et al., 1999; Li et al., 2003a; Wang et al., 2007a; Li et al., 2009; Li et al., 2012; Xin et al., 2014; Chen et al., 2020b). BFC has the effects of eliminating phlegm and antitussive, reducing fever, and it is used for the treatment of diseases associated with dry cough, chronic cough, cough with bloody sputum, consumptive cough, sores, swelling, and lung or breast abscesses (Wagner et al., 2011). In particular, BFC is effective for the treatment of the elderly and children, especially when it is difficult to recover after long-term treatment (Gao et al., 1999). Furthermore, BFC is stew with diets to nourish the lung against pulmonary diseases induced by particulate matter and smoking (Guo et al., 2017; Guo et al., 2020a). Therefore, BFC is regarded as a good medicine to promote lung health and has been utilized to treat pulmonary diseases in China and many other countries for thousands of years (Lin et al., 2015; Zhao et al., 2018a). Currently, there are more than 200 kinds of products related to BFC in the market such as Nin Jiom Pei Pa Koa, Chuanbei Zhike Lu, Chuanbei Pipa Capsules, etc., widely used to treat pulmonary diseases clinically, like dyspnea, bronchitis, chronic obstructive pulmonary disease (COPD), pulmonary tuberculosis, and lung cancer (Zhang et al., 2009; Li et al., 2013; Chinese Pharmacopoeia Com, 2015; Cunningham et al., 2018; Guo et al., 2020a) (Table 1).

**TABLE 1:** The main products related to BFC widely used to treat pulmonary diseases clinically

Name	Figure	Clinical usages	References
Nin Jiom Pei Pa Koa	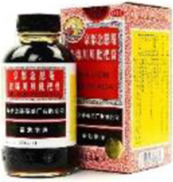	Sore throat	Drugs.com, (1064a)
Chuanbei Zhike Lu	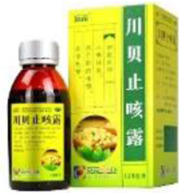	Dry cough, phlegm	Chinese Pharmacopoeia Com, (2015)
Chuanbei Pipa Tangjiang	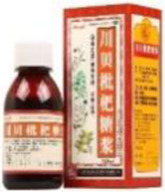	Cough, phlegm, sore throat, chest pain, cold, bronchitis	Chinese Pharmacopoeia Com, (2015)
Chuanbei Xueli Gao	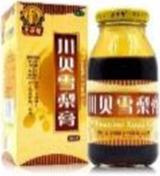	Cough, dyspnea, dry throat	Chinese Pharmacopoeia Com, (2015)
Niuhuang Shedan Chuanbei Ye	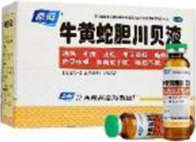	Phlegm, dry cough	Chinese Pharmacopoeia Com, (2015)
Zhike Chuanbei Pipa Diwan	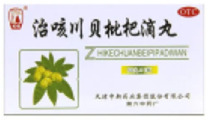	Cold, bronchitis, phlegm, cough, COPD.	(Chinese Pharmacopoeia Com, (2015); Xiao and Li, (2013))
Zhike Chuanbei Pipa Lu	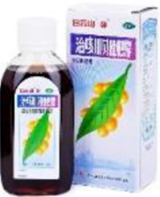	Cold, bronchitis, phlegm, cough	Chinese Pharmacopoeia Com, (2015)
Fufang Chuanbeijing Pian	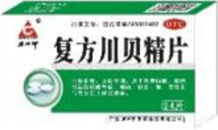	Cough, phlegm, asthma, acute and chronic bronchitis, chest distress	Chinese Pharmacopoeia Com, (2015)
Shedan Chuanbei Ruanjiaonang	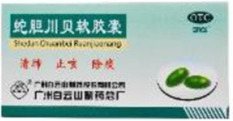	Cough, phlegm	Chinese Pharmacopoeia Com, (2015)
Shedan Chuanbei Jiaonang	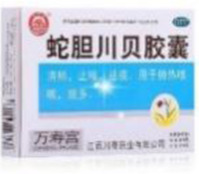	Cough, phlegm	Chinese Pharmacopoeia Com, (2015)
Shedan Chuanbei San	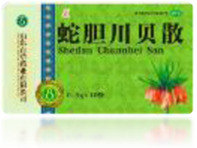	Cough, phlegm	Chinese Pharmacopoeia Com, (2015)
Chuanbei Pipa Capsules	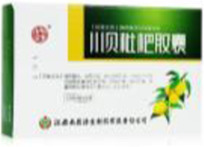	Cough, phlegm, chest pain, cold, sore throat, chronic bronchitis, COPD	Liu et al. (2019)
Feitai Capsule	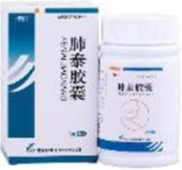	Cough, phlegm, fatigue, lung cancer, pulmonary tuberculosis	(Deng et al., 2012; Yu et al., 2012) (Liang et al., 2017)

So as for COVID-19, BFC can improve symptoms such as fever, dry cough, phlegm, shortness of breath, and sore throat (shown in Figure 1C). The chemical components of BFC are various and complex, including alkaloids, saponins, terpenoids, glycosides, nucleosides, nucleobases, fatty acids, and starches, among which alkaloids and saponins are considered to be the major low molecular-weight active components, especially alkaloids such as imperialine, chuanbeinone, verticine, verticinone, peimisine, isoverticine, delavine, delavinone, ebeiedinone, sipeimine, puqiedinone, puqiedine, peimisine-3-O-β-D-glucopyranoside, imperialine-3-β-D-glucoside, and so on (Kaneko et al., 1986; Ding et al., 1996; Li et al., 2006; Wang et al., 2007b; Duan et al., 2011; Zhang et al., 2011; Hao et al., 2013; Li et al., 2013; Lin et al., 2013; Peng et al., 2013; Wang et al., 2014a; Wang et al., 2017a; Geng et al., 2018; Wang et al., 2019; Chang et al., 2020). It is worth noting that verticine is also known as peimine and verticinone is also known as peiminine. Modern pharmacological studies have shown that the alkaloids in BFC have significant antitussive, expectorant, anti-asthmatic, anti-inflammatory, anti-oxidant, antitumor, and angiotensin converting enzyme (ACE) inhibition activities and exhibit good curative effect on cough, sputum, tracheobronchial contraction, acute lung injury, inflammation, and lung cancer (Luo et al., 2012; Wang et al., 2017b; Zhao et al., 2018b; Yin et al., 2019; Chen et al., 2020c). For example, imperialine is one of the essential steroidal alkaloids of BFC (Lin et al., 2013; Lin et al., 2015). Wang’s research (Wang et al., 2016a) found that in the COPD-like rat model associated with abnormal inflammatory response in the lung, imperialine could reduce the damage of lung function and structure. Moreover, imperialine could inhibit inflammation by regulating the expression of related cytokines such as IL-1β, IL-6, IL-8, TNF-α, NF-kB, TGF-β1, MMP-9, and TIMP-1. What’s interesting is that the COPD has influence on the progression and outcomes of COVID-19 (Wu et al., 2020c) and recent reports demonstrate that the majority of the serum levels of the above-mentioned cytokines and growth factors in COVID-19 patients will also increase (Chen, 2020; de la Rica et al., 2020; Nile et al., 2020; Phoswa and Khaliq, 2020; Wiersinga et al., 2020; Ghazavi et al., 2021; Metzemaekers et al., 2021). Besides, BFC exerts a significant therapeutic effect on various lung diseases, such as acute lung injury and lung inflammation. While severe acute lung injury is known as ARDS (Force et al., 2012) and lung inflammation is one of the characteristics of COVID-19.

The respiratory diseases that can be intervened by BFC are asthma, COPD, ARDS, lung cancer, and pulmonary tuberculosis. BFC is mainly playing a therapeutic role in lung diseases through the biological processes about inflammatory response, immune response, apoptosis, and oxidative stress. To date, there is no specific drugs for COVID-19, and the aim of this review is to summarize and analyze the pharmacological effects and mechanisms of BFC on respiratory pulmonary diseases, then provide insights on the drug discovery of anti-COVID-19 (Figure 1D).

## The Antitussive, Expectorant, and Anti-inflammatory Mechanisms of BFC

Cough is one of the common symptoms of various respiratory diseases, such as asthma, chronic bronchitis, pneumonia, and so on (Irwin and Madison, 2013). Although cough can be relieved by morphine containing codeine and other commonly used drugs, these drugs are highly addictive and cause side effects. BFC, as an antitussive TCM, has a positive therapeutic effect for cough due to its major bioactive alkaloids and it is non-addictive. Moreover, compared with synthetic drugs, BFC has fewer or no side effects (Zhang et al., 2009; Shang et al., 2010; Wu et al., 2018).

Xu (Xu et al., 2019) studied the antitussive, expectorant, and anti-inflammatory effect of BFC extract prepared through refluxing with 80% ethanol solvent administered orally to mice, respectively. The experiment of cough caused by ammonia was used to observe the antitussive effect of BFC. Phenol red expectoration test in mice trachea was conducted to investigate the effect of phlegm expelling. Auricular swelling model of mice was induced by xylene to research on the effect of BFC on lessening the ear swelling. The results demonstrated that BFC extract could obviously prolong the period of cough latency and inhibit the cough frequency of mice induced by ammonia. Moreover, the BFC extract could also significantly increase the output of phenol red in mice trachea and inhibit the ear swelling in anti-inflammatory experiment. After that, this research group (Wang et al., 2011) continued to study and find that the specific material basis for the antitussive effect of alkaloids was imperialine, chuanbeinone, verticinone, and verticine isolated from the BFC, which could markedly inhibit the cough frequency and prolong the latent period of cough in mice caused by ammonia. Besides, the specific material basis for the expectorant effect was imperialine, verticinone, and verticine, which could significantly increase the output of phenol red in mice trachea in the expectorant test. Then the specific material basis for the anti-inflammatory effect was imperialine and chuanbeinone, which could obviously inhibit the ear edema of mice in a dose-dependent manner. Therefore, the antitussive, expectorant, and anti-inflammatory bioactive components of BFC are the four alkaloids, imperialine, chuanbeinone, verticinone, and verticine, among which imperialine serves as the most critical role. The bulbs of *F. wabuensis* are also one of the sources of BFC. The team of Wang (Wang et al., 2012a) continued to study the antitussive, expectorant, and anti-inflammatory activities of BFC from *F. wabuensis* with the same models, respectively. The results indicated that all four alkaloids, imperialine, imperialine-β-N-oxide, isoverticine, and isoverticine-β-N-oxide isolated from BFC had significantly antitussive, expectorant, and anti-inflammatory effects, similarly. Besides, there were studies *in vivo* and *in vitro* that identified the antitussive effect of the fritillaria alkaloid crude extracts along with imperialine and among them imperialine was proved to be the most potent alkaloid (Chan et al., 2000). Zhang (Zhang et al., 2020a) investigated the potential molecular targets and mechanisms of verticine for cough through computational target fishing. The results of the study demonstrated that MAPK1, AKT1, and PPKCB were the key targets of verticine for treating cough. It was associated with the regulation of function for immunology and neurology exerting multi-proteins and multi-pathways effect (Figure 1C).

Inflammation is an automatic defense response of an organism to injury factors such as infection, noxious stimuli (chemicals), tissue injury, and so on. The typical inflammatory triggers are infection and tissue injury, which elicit the recruitment of leukocytes and plasma proteins to affected tissue sites (Galli et al., 2008; Medzhitov, 2008). Wang (Wang et al., 2016a) used different inflammatory animal models *in vivo* to evaluate the anti-inflammatory activity of purified total alkaloid fraction (TAF) from BFC, prepared by using H-103 resin column. Models of acetic acid-induced capillary permeability accentuation, and cotton pellet-induced granuloma formation were performed, respectively. His research demonstrated that TAF could inhibit acetic acid-induced capillary permeability accentuation, and cotton pellet-induced granuloma formation. It demonstrated that TAF had a strong anti-inflammatory effect. What is more, Wu (Wu et al., 2015) studied the anti-inflammatory activity and relative mechanisms of verticinone and imperialine, steroidal alkaloids from bulbs of *F. wabuensis* on LPS-stimulated RAW 264.7 macrophages. The research found that verticinone or imperialine could inhibit the production of NO, TNF-α, IL-1β, and the expressions of iNOS and COX-2. Meanwhile, they could decrease the phosphorylation of NF-κB in a dose dependent manner. The results of this study clearly demonstrated that the anti-inflammatory activity and mechanisms of verticinone and imperialine in BFC were associated with the inhibition of the activation of NF-κB signaling pathway. In addition, the research of Liu (Liu et al., 2020a) investigated the anti-inflammatory activity and its mechanisms of alkaloids from BFC in LPS-induced RAW264.7 macrophage cells. The results indicated that five of the alkaloids including imperialine, verticinone, verticine, peimisine, and delavine, could lower the production of NO, TNF-α, and IL-6, and inhibit the mRNA expressions of TNF-α and IL-6. The anti-inflammatory mechanisms of alkaloids from BFC were related to the inhibition of the phosphorylated activation of MAPK signaling pathways, and ERK1/2, p38 MAPK, and JNK/SAPK were included.

**FIGURE 2:**
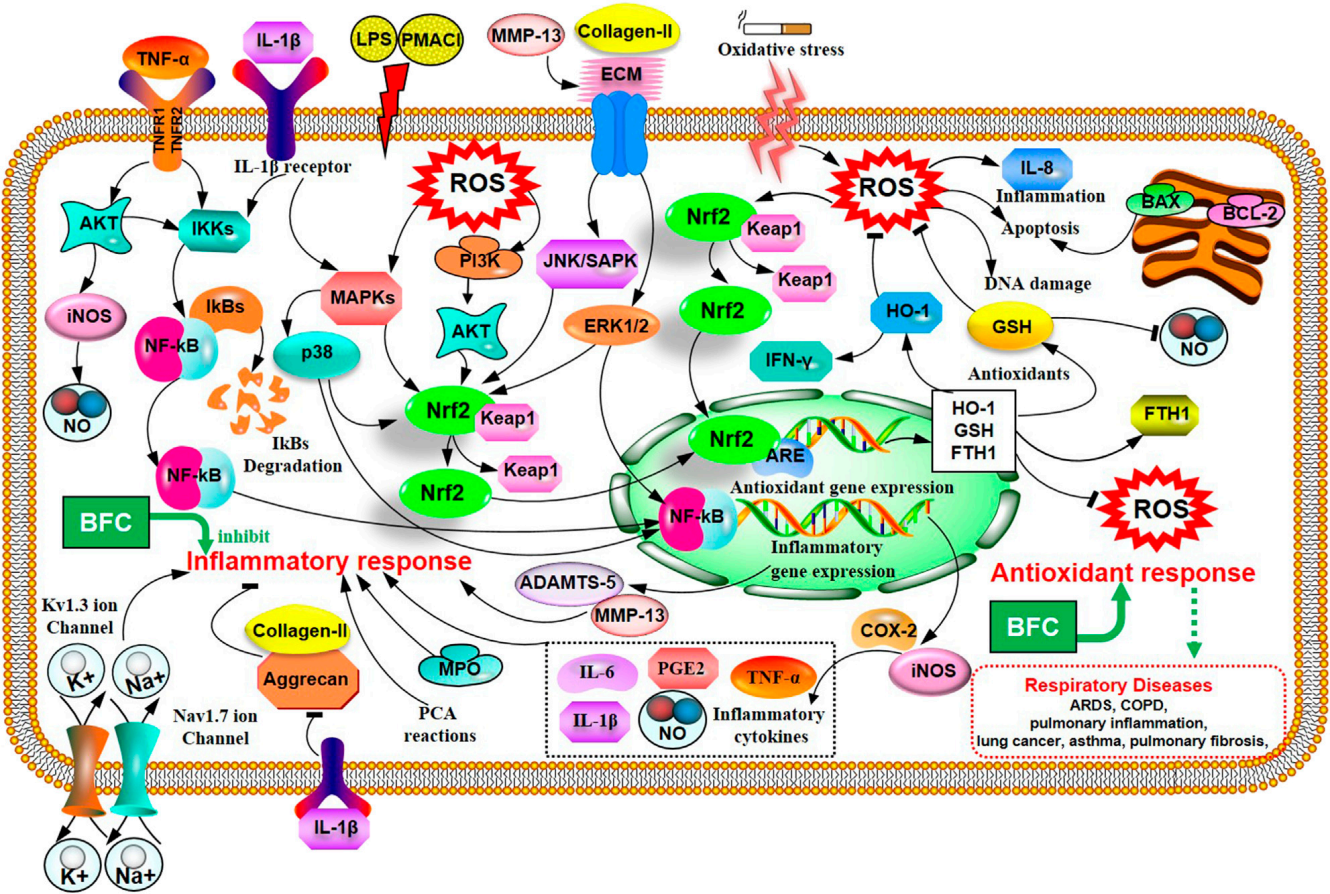
The anti-inflammatory and anti-oxidative stress signaling pathways of BFC.

In the study of Park (Park et al., 2017), it was proved that verticine was able to attenuate the production of pro-inflammatory cytokines IL-6, IL-8, and TNF-α and reduce the phosphorylation of MAPKs and the expression of NF-κB in PMACI-induced human mast cell (HMC-1). Verticine could also decrease the passive cutaneous anaphylaxis (PCA) reactions in rats. It suggested that verticine could be used to treat mast cell-derived allergic inflammatory reactions. The patch-clamp technique with HEK 293 cell lines was applied to study the anti-inflammatory and pain-relieving mechanisms of verticine against selected ion channels by Xu (Xu et al., 2016). The results of this research informed that the potential mechanisms of verticine for pain relieving and anti-inflammation were to inhibit Kv1.3 ion channel in a concentration dependent manner and block the Nav1.7 ion channel. The antinociceptive effect on inflammatory pain and paclitaxel induced cancer-related neuropathic pain of verticinone were also explored to be generally through both peripheral and central mechanisms in the rat models in Xu’s study (Xu et al., 2011). Luo (Luo et al., 2019a) evaluated the anti-inflammatory effect of verticine on IL-1β induced inflammatory response in mouse articular chondrocytes and ameliorates murine osteoarthritis model. The results showed that verticine could dramatically inhibit the expression of NO, PGE2, COX-2, TNF-α, iNOS, and IL-6 through pretreatment *in vitro*. Verticine was able to evidently increase the expression of aggrecan and collagen-II, alleviate the degradation of extracellular matrix (ECM) and reduce the production of thrombospondin motifs 5 (ADAMTS-5) and MMP-13 in a dose-dependent manner. The anti-inflammatory mechanisms of verticine were related to the inhibition of AKT phosphorylation and NF-κB activation with the activation of Nrf2/HO-1 signaling pathways. The anti-pulmonary inflammation and anti-pulmonary fibrosis activities of verticinone in rat models of bleomycin-induced lung injury were investigated by Guo (Guo et al., 2013). The data of the study informed that verticinone obviously ameliorated inflammation of alveolar and lung interstitial. Verticinone dramatically suppressed pulmonary fibrosis of bleomycin-induced rat model through down-regulating IFN-γ levels in serum and TGF-β, NF-κB, CTGF, ERK1/2, and FasL in pulmonary tissue markedly, which were comparable to dexamethasone. Gong (Gong et al., 2018) researched the anti-inflammatory properties of verticinone on LPS-induced mastitis model in mice and the mouse mammary epithelial cells (mMECs) model stimulated with LPS *in vitro* via being pretreated with verticinone. The data identified that verticinone exhibited potent anti-inflammatory effects on LPS-induced mastitis in mice, which could distinctly alleviate the histopathological injury of the breast *in vivo* and noticeably lower the MPO activity. Meanwhile, it was able to decrease the production of pro-inflammatory mediator TNF-α, IL-6, IL-1β, COX-2, and iNOS *in vivo* and *in vitro*. The anti-inflammatory mechanism of verticinone on mastitis was associated with the suppression for phosphorylation of AKT/NF-κB, ERK1/2, and p38 signaling pathways. These studies indicate that alkaloids from BFC have anti-inflammatory property, and BFC may be a great potential candidate to be developed for the prevention and treatment of inflammatory diseases. The anti-inflammatory mechanisms of BFC are shown in Figure 2.

## The Mechanisms of BFC in the Treatment of Asthma, COPD, and ARDS

Asthma is a long-term inflammatory disease of the airways, which is featured by overproduction of Th2 cytokines such as IL-4, IL-5, and IL-13 as well as accumulation of pulmonary eosinophils (Bousquet et al., 2000; Douwes et al., 2002; Factor, 2003; Yeum et al., 2007). BFC is a well-known TCM for the treatment of asthma and bronchial inflammation, and pharmacological studies have demonstrated that BFC has significant anti-asthmatic effect.

**FIGURE 3:**
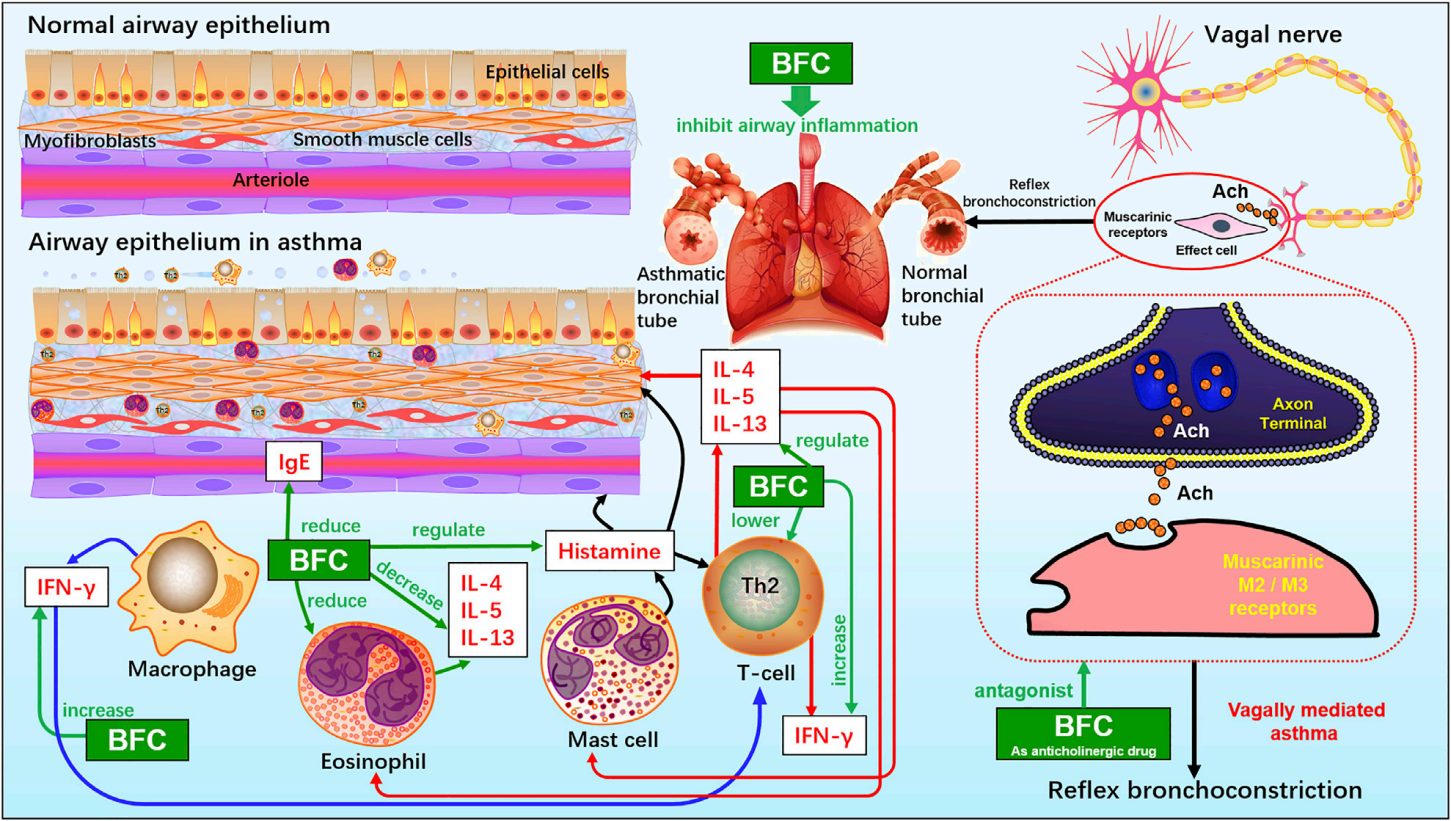
The molecular mechanism of BFC for the inhibitory effect on asthma and bronchial inflammation.

Yeum (Yeum et al., 2007) investigated the eosinophilic accumulation in the lungs, regulation of Th2 cytokine, and production of histamine and immunoglobulin E (IgE) in a murine model of asthma to research the anti-asthmatic effects of BFC. Eosinophilic proliferation was carried out by the uptake of [3H] thymidine, and accumulation of eosinophils. The study found that BFC significantly down-regulated the levels of IL-5, IL-13, and IL-4 in the bronchoalveolar lavage fluid and there was also a reduction of the level of ovalbumin-specific IgE in serum. BFC could lower the number of eosinophils by inhibiting the recruitment of eosinophil and airway inflammation. Therefore, it implies that BFC possesses a strong inhibitory effect on bronchial inflammation by reducing the production of Th2 cytokines such as IL-4, IL-5, and IL-13, IgE, histamine, and decreasing the accumulation of eosinophils and increasing the production of interferon-γ. The cholinergic nervous system is of significance in asthma and COPD. When the organism is infected by virus, or the antigen is inhaled, vagally mediated reflex bronchoconstriction occurs and the increase of reflex bronchoconstriction leads to asthma attacks. The dysfunction of suppressive muscarinic M2 receptors on the vagal nerve endings can enhance the release of acetylcholine. Therefore, the anticholinergic drugs may be of great benefit to treating acute asthma and these reflections can be interrupted effectively by the modified anticholinergics, such as selective M3 antagonists (Jacoby and Fryer, 2001; Lin et al., 2006; Gosens and Gross, 2018). Studies showed that the alkaloids in BFC had potential anti-asthmatic activities. Imperaline and sinpeinine A are the two alkaloids of BFC, and 3β-acetylimperialine is a derivative of imperialine. The research of Lin (Lin et al., 2006) found that imperaline and sinpeinine A were the antagonists of selective muscarinic M2 receptor subtype, and 3β-acetylimperialine was a selective muscarinic M3 receptor subtype antagonist at the cell level. Moreover, in tracheal smooth muscle the spasmolytic effects of imperialine and sinpeinine A were weaker than 3β-acetylimperialine. It suggested that the effective compounds of BFC for the treatment of asthma were 3β-acetylimperialine, imperaline, and sinpeinine A. The mechanism of anti-asthmatic effect of BFC may be related to the selective antimuscarinic activity. In addition, the anticholinergic activities of imperialine were compared with its derivatives, imperialinol, 3β-acetoxyimperialine, 3β-propionoxyimperialine, and 3β-butyroxyimperialine to reveal the structure-activity relationship by Rahman (Atta-ur-Rahman et al., 1998). It was shown that 3β-propionoxyimperialine and 3β-butyroxyimperialine exerted better anticholinergic effect against muscarinic receptors of the heart and brain than imperialine but imperialinol and 3β-acetoxyimperialine exhibited less effective. Therefore, these researches indicate that the alkaloids along with their derivatives may be promising anti-asthmatic candidates in the future and some derivatives are more potent and the mechanisms of BFC for the inhibitory effect on asthma and bronchial inflammation are shown next (Figure 3).

Liu (Liu et al., 2020b) studied the impacts of six isosteroid alkaloids from BFC on oxidative stress induced by cigarette smoke (CS) and its protection mechanism in RAW264.7 macrophages, in which the alkaloids, verticinone, verticine, imperialine, imperialine-3-β-D-glucoside, delavine, and peimisine were included. The study indicated that the alkaloids in BFC could down-regulate the production of ROS and up-regulate the level of GSH and expression of HO-1 and Nrf2. Furthermore, among them, the effect against cigarette smoke extract (CSE)-induced oxidative stress of imperialine was weaker than that of verticinone, verticine, imperialine-3-β-D-glucoside, delavine, and peimisine. The results elucidated that the property for anti-oxidative stress of alkaloids in BFC and their protective effect against oxidative stress were through activating antioxidant signaling pathway of Nrf2. It identified that BFC as an inhibitor of oxidative stress might be a promising therapeutic drug for diseases related to oxidative stress. Oxidative stress is associated with the pathogenesis of various respiratory diseases, such as ARDS, pulmonary inflammation, lung cancer, asthma, pulmonary fibrosis, and COPD (Bargagli et al., 2009; Kirkham and Barnes, 2013; Valavanidis et al., 2013; Anathy et al., 2018; Gonzalez-Juarbe et al., 2020). A study showed that the activation of Nrf2-mediated antioxidant signaling pathway in asthma mouse model could attenuate ROS-induced airway remodeling (Zeng et al., 2019). Therefore, BFC has the potential therapeutic effects in asthma through anti-oxidation. The anti-oxidative stress mechanisms of BFC are shown above (Figure 2).

**FIGURE 4:**
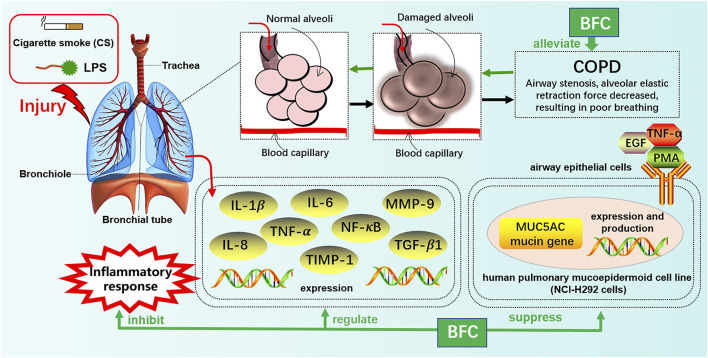
The mechanism of BFC in the treatment of COPD.

Furthermore, Kim’s study (Kim et al., 2016) investigated the effects of verticine on EGF, PMA, or TNF-α induced MUC5AC mucin gene expression and production in human pulmonary mucoepidermoid cell line, NCI-H292 cells. The results revealed that verticine could suppress MUC5AC mucin gene expression and production via acting upon airway epithelial cells directly, which suggested that verticine might be suitable for diverse pulmonary inflammatory diseases. COPD is one of the leading causes of death ranking third worldwide. Wang’s research (Wang et al., 2016a) explored the effect of imperialine, one of the essential steroidal alkaloids from BFC, on the function, structure, and inflammation of the lung in a COPD-like rat model induced by the combination of exposure to CS and intratracheal administration of LPS. The results demonstrated that imperialine could alleviate the injury of lung function and structure to reduce the progression of COPD. Imperialine exhibited an inhibition of inflammatory response in the lung by regulating the expression of IL-1β, IL-6, IL-8, TNF-α, NF-kB, TGF-β1, MMP-9, and TIMP-1, which were correlative cytokines in pulmonary tissues. The TNF signaling pathway exhibits an essential role in inflammation, cell proliferation, and cell death. Dysregulation of signal transduction of NF-κB induced by TNF receptor 1 (TNFR1) leads to chronic inflammation, which is related to a variety of human inflammatory pathology, such as ARDS, pulmonary vascular endothelial injury, and pulmonary interstitial inflammation (Stephens et al., 1988; Goldblum et al., 1989; Chen and Goeddel, 2002; Kalliolias and Ivashkiv, 2015; Van Quickelberghe et al., 2018; Wang et al., 2018). The mechanisms of BFC in the treatment of COPD are shown in Figure 4.

**FIGURE 5:**
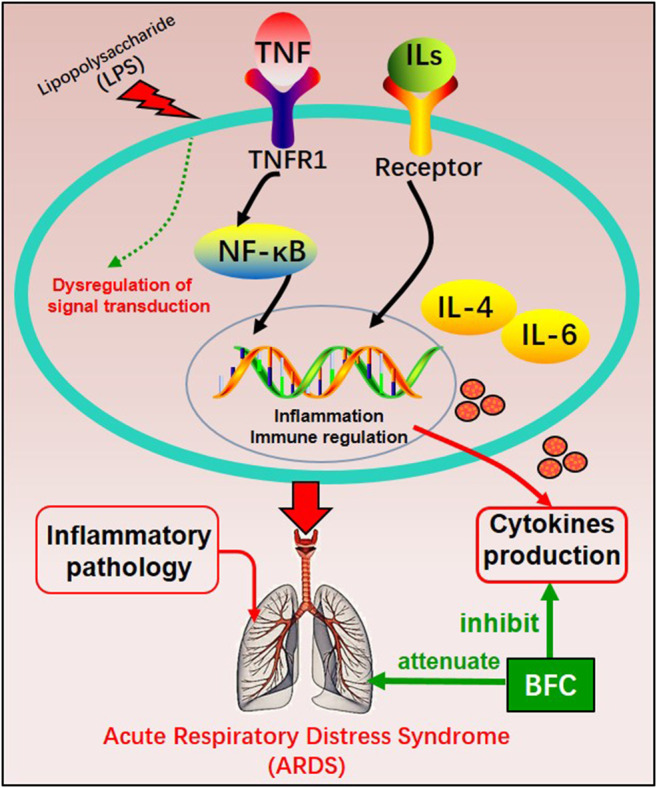
The mechanism of BFC in the treatment of ARDS.

The study of Wang (Wang et al., 2016) investigated the anti-inflammatory effect in the lung of alkaloids in BFC *in vivo* by using lipopolysaccharide (LPS)-induced ARDS. The results showed that alkaloids in BFC could inhibit inflammatory cells recruitment and cytokines production such as TNF and IL-6 in the bronchoalveolar lavage fluid from LPS-induced ARDS mice, and attenuate pathological changes in the lung tissues. The mechanisms of BFC in the treatment of ARDS are shown in Figure 5. As for lung injury, it is characterized by pulmonary inflammatory reaction and will develop into pulmonary fibrosis with deposition of fibrin and collagen (Limper, 2004; Graves et al., 2010; Bhatia et al., 2012). These studies suggested that BFC showed a better therapeutic effect on pulmonary inflammatory diseases such as asthma, COPD, and ARDS.

## The Mechanisms of BFC in the Treatment of Lung Cancer

Lung cancer is one of the most frequent cancers, remaining the leading cause of cancer death among males globally (Bray et al., 2018) and the pathogenesis of cancer is bound up with proliferation, apoptosis, inflammation, etc. (Xiu et al., 2015). Lung cancer comprises small-cell lung cancer (SCLC) and non-small cell lung cancer (NSCLC), among which SCLC accounts for approximately 15% of all lung cancers and NSCLC accounts for approximately 85% including adenocarcinoma, squamous-cell carcinoma, and large-cell carcinoma (Oser et al., 2015; Remark et al., 2015; Reck et al., 2017; Herbst et al., 2018; Osmani et al., 2018; Yang et al., 2019). BFC displays significant anti-tumor activity, and its extract as well as alkaloids exhibit anti-proliferative effects on the growth of glioblastoma, colorectal, oral, human promyelocytic leukemia, immortalized keratinocyte, hepatoma, Ehrlich ascites carcinoma, ovarian, and endometrial cancer cells (Ping et al., 1995; Pae et al., 2002; Yun et al., 2008; Bokhari and Syed, 2015; Kavandi et al., 2015; Zheng et al., 2017; Zhao et al., 2018b). Bokhari (Bokhari and Syed, 2015) found that BFC could significantly suppress TGF-β/SMAD signaling pathway to inhibit cancer cell proliferation, invasion, and metastasis. What’s more, studies showed that BFC exerted a potent therapeutic effect on lung cancer (Wang et al., 2014b; Chen et al., 2020d). Li suggested that BFC was the most commonly used single herb for the treatment of lung cancer (Li et al., 2018). BFC is contributing to the treatment and survival of lung cancer patients in clinical practice, and it is widely used as an adjuvant treatment of lung cancer chemotherapy in TCM (Kavandi et al., 2015), even to the extent that BFC can further enhance the treatment efficiency of lung cancer from 70 to 95% (Lin et al., 2020).

In the study of Li (Li et al., 2020c), the effects of aqueous extract of BFC (AE) along with its mechanism in NSCLC A549 cells *in vitro* and xenograft model of nude mice *in vivo* were assessed. The results *in vitro* revealed that AE displayed inhibition of A549 cells proliferation and colony formation and promotion of apoptosis. RNA-seq was performed using GO and KEGG pathway enrichment analysis and it was found that the dominant differentially expressed genes (DEGs) were associated with apoptosis, immune response, and cell cycle process. While AE increased the expressions of STAT 1 and STAT4 as well as their target genes IFN-γ and IL-12, triggered Bcl-2/Bax proteins attributing to cellular apoptosis in A549 cells. The results *in vivo* showed that AE lessened the size of tumor and induced cytokines IL-12 and IFN-γ secretion. BFC showed notably antitumor activity through a co-regulatory network mediated by STAT1 and STAT4 for activation of immunomodulation to induce apoptosis. Wang (Wang et al., 2014b) used the human lung carcinoma cell line (A549) to investigate the antitumor activity of BFC *in vitro*, in which the antiproliferative activities of the different fractions from BFC including MeOH extracts (ME), petroleum ether extracts (PE), chloroform extracts (CE), n-hexane extracts (HE), water extracts (WE), and the purified total alkaloids of BFC (TAF) were to be examined. The results of the study demonstrated that CE, primarily containing total alkaloids and the TAF displayed higher antiproliferative effect than the others. The major alkaloids monomers in CE and TAF were peimisine, imperialine, and chuanbeinone and TAF displayed obviously antineoplastic activity and low toxicity *in vivo*. The antitumor mechanism of TAF was to suppress tumor angiogenesis and promote apoptosis through increasing the level of caspase-3 expression.

Wang (Wang et al., 2014c) similarly made an investigation of antitumor activities of BFC extracts against Lewis lung carcinoma cells (LLC). The different fractions from BFC were ME, PE, CE, HE, TAF, and WE. It was indicated that CE and TAF displayed stronger suppression of proliferation on LLC cells than others. The three dominant alkaloid compounds (peimisine, imperialine, and chuanbeinone) in CE and TAF could significantly inhibit the proliferation of LLC cells. The inhibitory effect against LLC cells growth of imperialine was weaker than chuanbeinone and peimisine *in vitro*. The cell cycle and sub-G1 group of LLC cells were assessed to find that the TAF could induce apoptosis and the cell cycle arrest. Moreover, the mice models were constructed by inoculating LLC cells suspension into the left armpit of C57BL/6 J mice subcutaneously with LLC cells maintaining in solid form for serial transplantation. The results *in vivo* indicated that TAF exerted antitumor effect obviously with low toxicity and could dose-dependently suppress the growth of transplantable LLC tumor. The antitumor mechanism of TAF was associated with the inhibition of tumor angiogenesis and promotion of apoptosis by activating caspase-3. Chuanbeinone showed obvious antitumor activity against LLC *in vitro* and induced S phase arrest and apoptosis of LLC in Wang’s other study (Wang et al., 2015a). Chuanbeinone decreased the antiapoptotic Bcl-2 expression and increased the proapoptotic protein Bax and caspase-3 expression. Moreover, chuanbeinone could suppress tumor angiogenesis and increase apoptosis by up-regulating caspase-3 expression *in vivo*, in which the LLC cells were subcutaneously inoculated into the left armpit of the mice. Verticine was also reported to suppress human lung adenocarcinoma A549/DDP cell proliferation dose-dependently and reverse multidrug resistance (MDR). The anti-lung cancer mechanisms of verticine were associated with apoptosis induction along with decreasing of lung resistance protein (LRP) and excision repair cross-complement 1 (ERCC1) mRNA expression (Yin et al., 2019). Imperialine, as quality control component (Ye and Wang, 2014), is one of the active compounds in alkaloids from BFC and is also the anti-inflammatory agent. The research of Lin (Lin et al., 2020) investigated the anti-cancer effects against NSCLC and its related molecular mechanism of imperialine *in vitro* and *in vivo*. A549 cell lines were used for NSCLC cells model *in vitro* and A549 tumor-bearing mouse model was built for *in vivo* research. It was found that the imperialine could significantly suppress the activity of NF-κB to inhibit not only NSCLC tumor but also inflammation both *in vitro* and *in vivo* through the inflammation-cancer feedback loop with extremely low toxicity and side effects on blood cell and the main organs. The NSCLC-targeting liposomal system was effectively developed for targeted drug delivery, which could promote the cellular uptake of imperialine at tumor sites and the accumulation *in vivo* to enhance the overall therapeutic effect of anti-tumor. Hence, these studies indicate that the alkaloids of BFC exert anti-cancer activity against lung cancer while exhibiting systemic safety, and BFC is expected to become a promising novel anti-tumor agent for lung cancer.

The STAT family of proteins is the central in modulating immune responses in the carcinoma microenvironment to accelerate or suppress malignant tumors by regulating cytokine-dependent inflammation and immunity, which are closely relevant to human cancer (Yu et al., 2009; Gutiérrez-Hoya and Soto-Cruz, 2020; Verhoeven et al., 2020). Apoptosis is a form of programmed cell death, which is a natural manner to efficiently eliminate the aged cells or damaged cells from the body. Hence, currently most anti-cancer drugs are inducing apoptosis and triggering correlative cell death signaling pathways to get rid of carcinoma cells (Mohammad et al., 2015; Pistritto et al., 2016; Mortezaee et al., 2019). The caspase family is recognized as a key participant in the execution of apoptosis, especially caspase-3 (Cohen, 1997; Anjum et al., 1998; Green and Llambi, 2015; Nagata, 2018; Ramirez and Salvesen, 2018). Transcription factor family NF-κB has been considered the central mediator in inflammation processes and an essential role in innate and adaptive immunity responses. The activation of NF-κB is also prevalent in cancer, in which NF-κB activation is primarily triggered by inflammatory cytokines within the carcinoma microenvironment. Furthermore, the survival genes in malignant tumor cells and the proinflammatory genes in carcinoma microenvironment are activated by NF-κB conversely (DiDonato et al., 2012; Fan et al., 2013; Hoesel and Schmid, 2013; Shostak and Chariot, 2015). Therefore, NF-κB plays a crucial role in inflammation and cancer.

**FIGURE 6:**
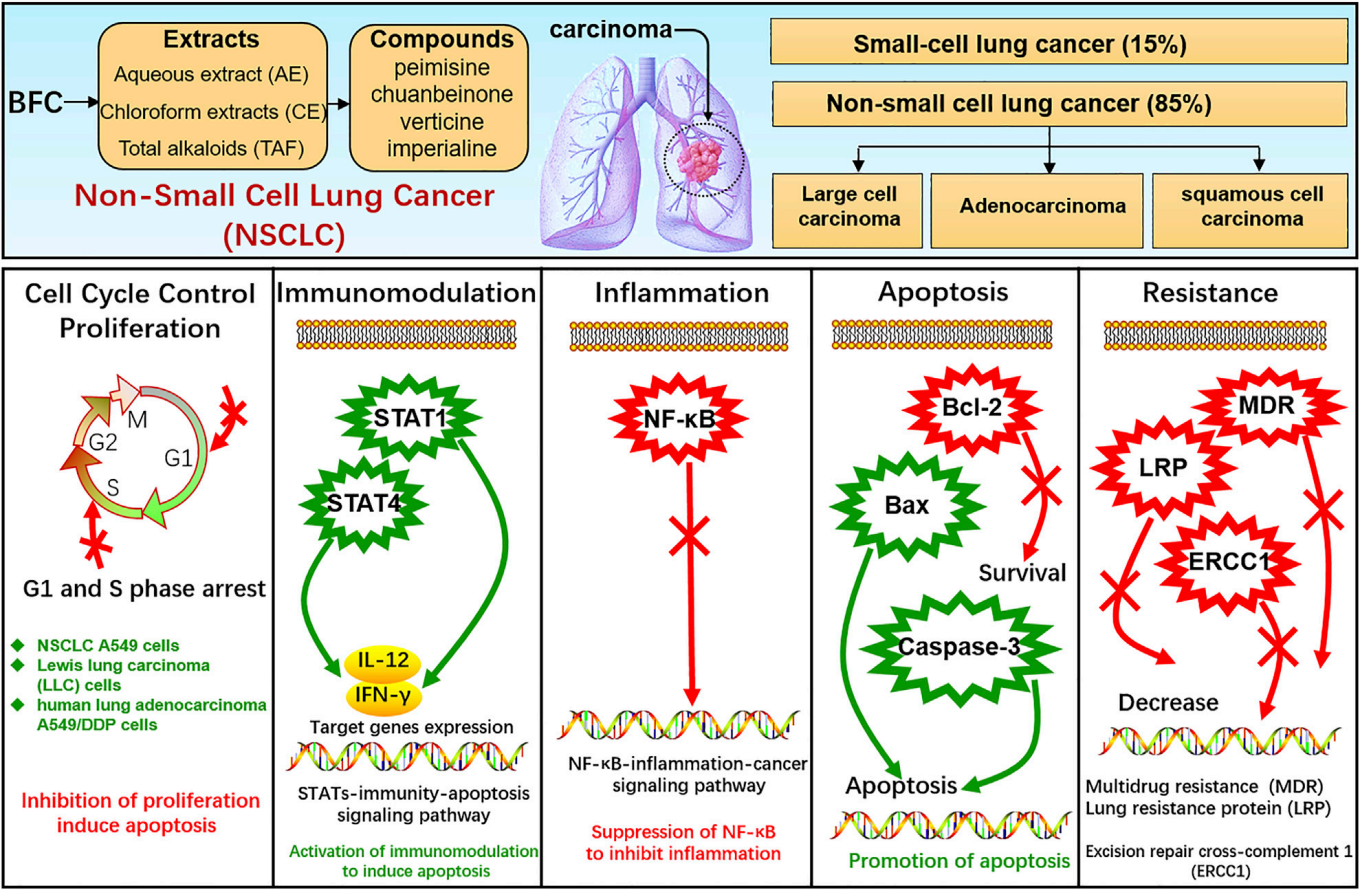
The mechanism of BFC in the treatment of lung cancer.

In this study, it is found that BFC has strong anti-tumor activity, exhibiting anti-proliferative effect on the growth of lung cancer cells, and BFC displays therapeutic effect on lung cancer *in vitro* and *in vivo*. The anti-lung cancer mechanisms of BFC include inducing apoptosis, anti-proliferation, preventing tumor invasion and metastasis, activating STATs-mediated immunomodulation to trigger apoptosis, inhibiting NF-κB-mediated inflammation-cancer feedback loop, up-regulating proapoptotic protein Bax and caspase-3, down-regulating antiapoptotic Bcl-2, reversing MDR, and decreasing LRP and ERCC1. It is of great significance to study the anti-lung cancer mechanism of BFC for its rational application and development. These results indicate that BFC may be a promising medication for lung cancer, which can be used as a new source of NF-κB inhibitor in the treatment of lung cancer. It also can be applied in modulating immune response to improve anti-lung cancer therapies and even to become an effective caspase-3 promoter in apoptosis signaling pathways. Besides, BFC can also be widely used in the adjuvant treatment of lung cancer owing to its extremely low adverse reactions and side effects, in order to ultimately improve the quality of life and prognosis for the lung cancer patients. The mechanisms of BFC in the treatment of lung cancer are shown in Figure 6.

## The Mechanisms of BFC in the Treatment of Pulmonary Tuberculosis

Tuberculosis (TB) is an infectious disease caused by bacteria *Mycobacterium tuberculosis* spread by coughing, speaking, or singing, which is still one of the top 10 causes of death (ranking above HIV/AIDS) and the leading cause of death from a communicable disease among adults in the world, with more than 10 million people getting ill due to tuberculosis every year (WHO, 2020). The pathological features of tuberculosis are typically pulmonary necrotizing granulomatous inflammation, namely the lungs are affected, which is called pulmonary tuberculosis (Wang et al., 2014d; Furin et al., 2019). The *Mycobacterium tuberculosis* in the lungs can also infect other parts of the body, such as the kidney, spine, and brain through the blood and approximately all external parts of the lung may be affected, which is called extrapulmonary tuberculosis (Dheda et al., 2016). Tuberculosis can affect anyone anywhere; therefore, it remains one of the greatest health issues worldwide.

TCM plays a crucial role in the treatment of tuberculosis and promotes the discovery and development of new anti-tuberculosis drugs (Wang et al., 2015b) and BFC has the action of anti-tuberculosis (Khare and Khare, 2007). Studies found that BFC in modern and ancient prescriptions could help improve the treatment of tuberculosis (Xu et al., 2020b). Feitai Capsule is made up of several Chinese herbal medicines, such as BFC, Stemonae Radix, Eriobotryae Folium, and Scutellariae Radix. Wang (Wang et al., 2017c) used Feitai Capsule to evaluate the clinical efficacy for retreated pulmonary tuberculosis. The results showed that Feitai Capsule combined with anti-tuberculosis chemotherapy drugs in the treatment of retreated pulmonary tuberculosis could effectively promote the absorption of the lesions and the closure of cavities and accelerate the conversion of sputum bacteria to negative. It was shown that Feitai Capsule could enhance the immunity and promote the inflammation dissipation of patients with pulmonary tuberculosis. The TCM Niubeixiaohe is composed of six kinds of TCM such as BFC, Rhizoma Bletillae, Radix Platycodonis, Fructus Arctii, and so on. Liang (Liang et al., 2017) investigated the effects of Niubeixiaohe powder and Niubeixiaohe extracts on tuberculosis mice models infected with *Mycobacterium tuberculosis* H37Rv *in vivo*. It was found that Niubeixiaohe powder and Niubeixiaohe extracts exhibited anti-tuberculosis effects and in particular the Niubeixiaohe extracts could distinctly improve the pulmonary lesions to return to normal pulmonary structure. It could be concluded from this research that Niubeixiaohe was an effective anti-tuberculosis prescription to treat tuberculosis in clinical practice.

**FIGURE 7:**
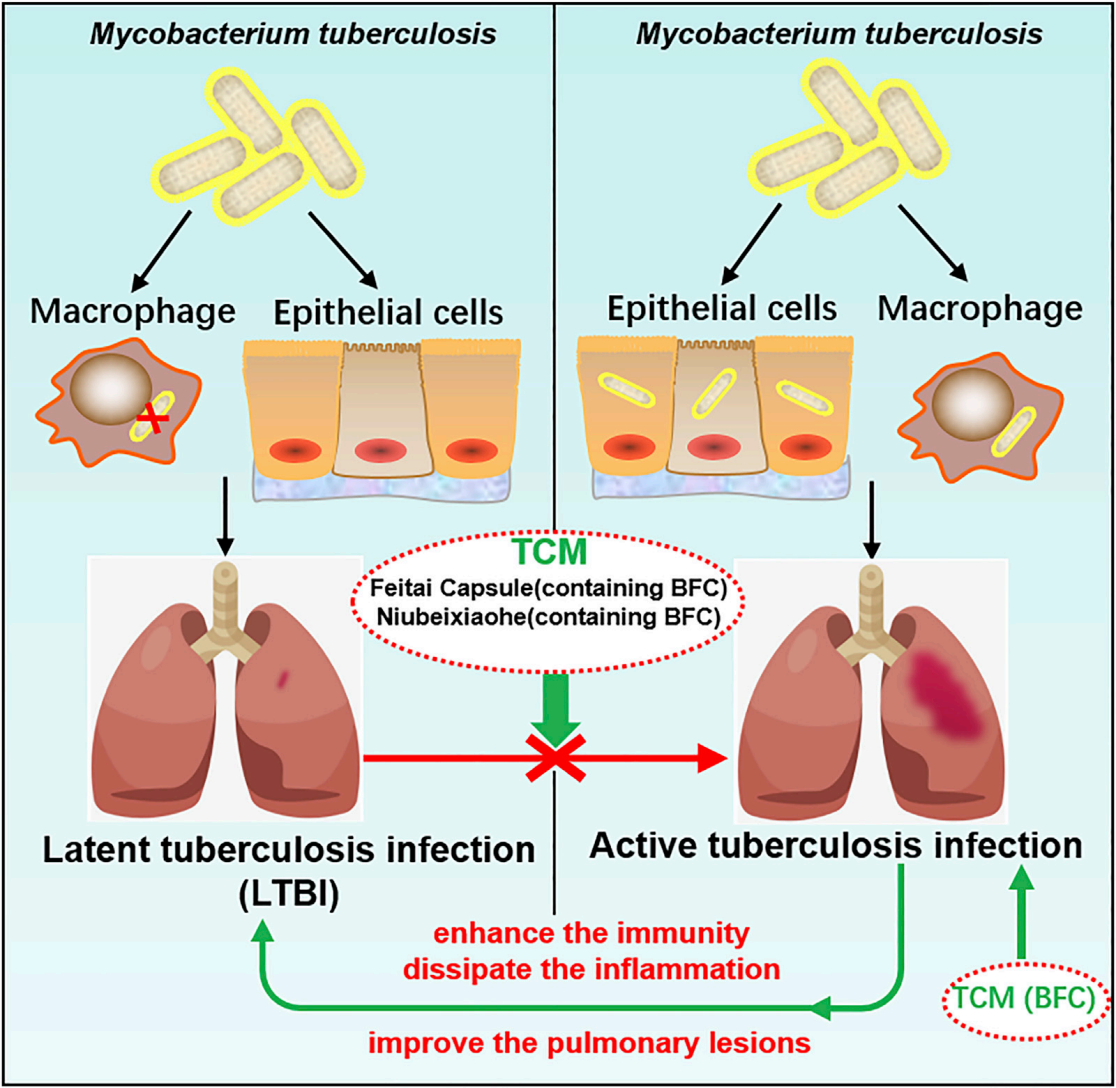
The mechanism of BFC in the treatment of pulmonary tuberculosis.

Latent tuberculosis infection (LTBI) is a condition of sustained immunity reaction to irritation by antigens of *Mycobacterium tuberculosis* with no phenomenon of active tuberculosis disease in clinical manifestation (Centers for Disease Control and Prevention [CDC]). Mostly there are no signs or symptoms of tuberculosis disease with no infectivity, but once triggered by some factors, it will develop into active tuberculosis disease and become infectious. The most important factor for active tuberculosis disease after infection is immunological situation (Falzon et al., 2020), combined with the findings of this study that BFC had a good protective effect on lung health, which displayed a strong role in activation of immunomodulation and suppressed lung inflammation. TCM prescriptions containing BFC had a good therapeutic effect on pulmonary tuberculosis. Therefore, BFC is a potential drug for the treatment of pulmonary tuberculosis and LTBI can be prevented effectively by making use of BFC, so that it does not become active tuberculosis disease. The mechanisms of BFC in the treatment of pulmonary tuberculosis are shown in Figure 7.

## Is There a Potential Drug in the Treatment of COVID-19?

**FIGURE 8:**
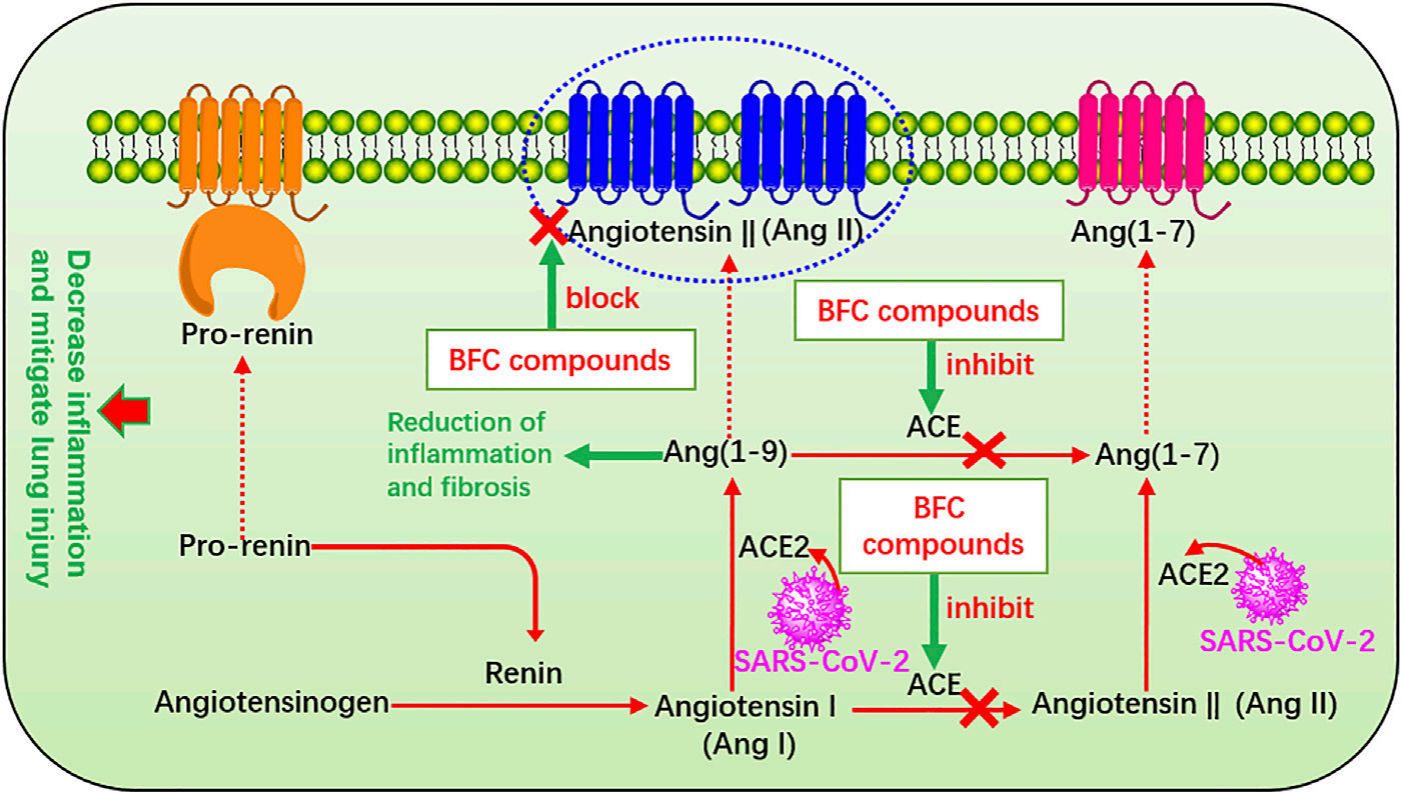
The renin-angiotensin system (RAS) that BFC compounds influence.

The renin-angiotensin system (RAS) plays a major role in control of electrolyte homeostasis, fluid, and blood pressure. Renin and angiotensin I converting enzyme (ACE) cleave the angiotensinogen, the major precursor protein of RAS into angiotensin I (Ang I) and angiotensin II (Ang II), respectively (Pahlavani et al., 2017). RAS consists of ACE, angiotensin converting enzyme 2 (ACE2), Ang II, and Ang II type 1 receptor (Xia and Lazartigues, 2010). The spike glycoprotein (S protein) on the surface of SARS-CoV-2 is a crucial structural protein. It is a highly glycosylated homotrimer and can mediate the invasion of SARS-CoV-2 into human cells (Zheng et al., 2020a; Xu et al., 2020c; Walls et al., 2020; Wrapp et al., 2020; Zhong et al., 2020). ACE2 is expressed in many other organs such as lung, stomach, kidney, brain, heart, spleen, liver, and intestines, among which the lung and small intestine are the main vulnerable parts (Hamming et al., 2004; Elased et al., 2008; Chen et al., 2020e) with the expression on the surface of many cell membranes like pulmonary alveolar, tracheal, and bronchial epithelial cells, and also the macrophages (Kai and Kai, 2020). ACE2 regulates the RAS by degradation of Ang II produced by ACE, and it is an important regulator of ARDS (Kai and Kai, 2020). ACE2 is identified as the host cell functional receptor of SARS-CoV-2 as well as SARS-CoV and virus enters the host cells by binding the S protein to ACE2 receptor and adsorbing onto it (Wu et al., 2020d; Gheblawi et al., 2020; Hoffmann et al., 2020). When SARS-CoV-2 enters the host, it will replicate, assemble, and release a great quantity of viral particles thus numerous viruses invade the body and cause disease. SARS-CoV-2 utilizes the identical cell entry receptor to SARS-CoV. That is to say, the two virus SARS-CoV-2 and SARS-CoV both use their expressed S protein to bind to ACE2 and enter the host cells. But the affinity of SARS-CoV-2 for ACE2 is 10–20 times that of SARS-CoV, which improves its transmission ability (Li et al., 2020b). Significantly, ACE is markedly different from ACE2. ACE also targets on Ang I to generate Ang II involved in the pathogenesis of ARDS, which leads to vasoconstriction and bronchoconstriction, increases blood vessel permeability, and triggers inflammation, fibrosis, and apoptosis. Thus it accelerates the progress of ARDS and lung failure in COVID-19 patients and SARS-CoV infection patients (Wang et al., 1999; Li et al., 2003b; Suzuki et al., 2003; Wösten-van Asperen et al., 2008; Rossi et al., 2020). However, ACE2 targets on Ang II to generate Ang (1–7), which improves ARDS and ACE2 gene deletion aggravates ARDS (Imai et al., 2005) and thus enzyme ACE2 provides a new therapeutic method for the syndrome (Wösten-van Asperen et al., 2011). ACE2 converts Ang I to Ang (1–9) (Donoghue et al., 2000) and Ang (1–7) and Ang (1–9) will result in reduction of inflammation and fibrosis (Wigén et al., 2020). Studies confirmed that it could decrease inflammation and mitigate lung injury through inhibiting ACE, or blocking the Ang II receptor in ARDS and the risk could not be increased by administration with ACE inhibitors after being infected by SARS-CoV-2, even more ACE inhibitors can increase ACE2 (Hagiwara et al., 2009; Wösten-van Asperen et al., 2010; Wysocki et al., 2020). Preoperative serum ACE activity was suggested as a useful prognostic indicator in lung cancer (Danilov et al., 2019). Therefore, the RAS plays an important part in the process of COVID-19 and other pulmonary diseases, and quickly it becomes a hot spot to apply RAS blockers to treat COVID-19 (Danser et al., 2020; Fang et al., 2020; Grasselli et al., 2020; Vaduganathan et al., 2020). In Oh’s study, it was found that verticinone, verticine, and peimisine could inhibit the activity of ACE in a dose-dependent manner, exhibiting as high as 50% inhibitory concentration of 165.0, 312.8, and 526.5 μM, respectively (Oh et al., 2003). Moreover, many other Fritillaria alkaloids were also found to have inhibitory activity against ACE significantly (Kang et al., 2002; An et al., 2010). The RAS that BFC compounds influence is shown in Figure 8.

Second, the systemic symptoms of COVID-19 patients showed that the levels of C-reactive protein, D-dimer, lactic dehydrogenase (LDH), leukocyte, and neutrophil-lymphocyte ratio (NLR) were elevated but the levels of lymphocytes and platelets were decreased (Chan et al., 2020; Guan et al., 2020; Henry et al., 2020; Qin et al., 2020; Çolak et al., 2021). In severe COVID-19 patients there was dramatically lower number of CD4^+^ and CD8^+^ T cells in the peripheral blood accompanied by the reduced lymphocytes in the spleen and lymph nodes as well as the lymphocyte degeneration, necrosis (Li et al., 2020b). Xiong (Xiong et al., 2020) found that the reason for patients’ lymphopenia might be associated with activation of apoptosis and P53 signaling pathway in lymphocytes induced by SARS-CoV-2. Interestingly, with the increasing age the function of naive CD4^+^ and CD8^+^ T cells may be damaged, which should be responsible for declined immunoregulation in the elderly (Rane et al., 2018; Li et al., 2019) and it might be one of the reasons why the severity is worse in older COVID-19 patients. Thus, the SARS-CoV-2 infection will trigger an immune response and mainly impact on T lymphocytes, especially CD4^+^ and CD8^+^ T cells, and potential immunological markers are related to COVID-19 (Chen et al., 2020f). Initially, adaptive immunoreaction is generated to purge the virus and halt disease process in the immune cell response (Shi et al., 2020). If the protective immunoreaction is damaged, the virus will replicate and impair tissue, particularly in apparatus with more expression of ACE2, just like lungs. The impaired pulmonary cells lead to innate inflammation, which is mainly arranged by macrophages and granulocytes mediating inflammation. With the aggravation of the disease, the potentially lethal lung inflammation occurs, accompanied by the appearance of high fever and myalgia (De Virgiliis and Di Giovanni, 2020), suggesting a serious immune imbalance. Consequently, the systemic immune system is abnormally activated and dysregulated and this immune response results in the cytokine release syndrome (CRS), namely “cytokine storm” that is an excessive immune response (Sinha et al., 2020; Ye et al., 2020). In the “cytokine storm” of SARS-CoV-2 infection there is an intense inflammatory response with the release of abundant increased pro-inflammatory cytokines such as IL-1β, IL-7, IL-8, IL-9, IL-10, FGF, G-CSF, GM-CSF, IFN-γ, IP-10, MCP-1, MIP-1A, MIP1-B, PDGF, TNF-α, and VEGF in both ICU and non-ICU COVID-19 patients (Liu et al., 2020c; Ragab et al., 2020). Along with the “cytokine storm” expanding, the monocytes/macrophages exhibit overactivation followed by acute lung injury, contributing to ARDS (Zhang et al., 2020b), which are the important factors of the COVID-19 transition from mild to severe diseases. Therefore, due to the reduced CD4^+^ and CD8^+^ T cells, the “cytokine storm” is mediated by leukocytes other than T cells and severe COVID-19 is related to excessive immune response bound up with CRS such as high levels of CXCL-10, CCL-7, and IL-1, which then result in loss of lung function, lung tissue injury, repair imbalance, and respiratory failure (Vaninov, 2020). Furthermore, the viral infection-immunity-inflammation of SARS-CoV-2 are throughout the whole process of COVID-19 even in the recovery stage or after hospital discharge (Wen et al., 2020). A report showed that through immunohistochemistry the expression of IL-4 and M2 macrophages scores of lung tissue were markedly increased in the patients with COVID-19, accompanied by the higher participation of the Th2 (Vaz de Paula et al., 2020). Hence, effective immunization and inflammation therapy can commendably reduce the transition from initial and advanced stages to severe and critical illness, and decrease the incidence of critical illness to lower the mortality. BFC was confirmed to possess a strong inhibitory effect on immunity-inflammation by reducing the production of Th2 cytokines such as IL-4 and IL-13, as well as acting on antagonists of selective muscarinic M2 receptor subtype.

**FIGURE 9:**
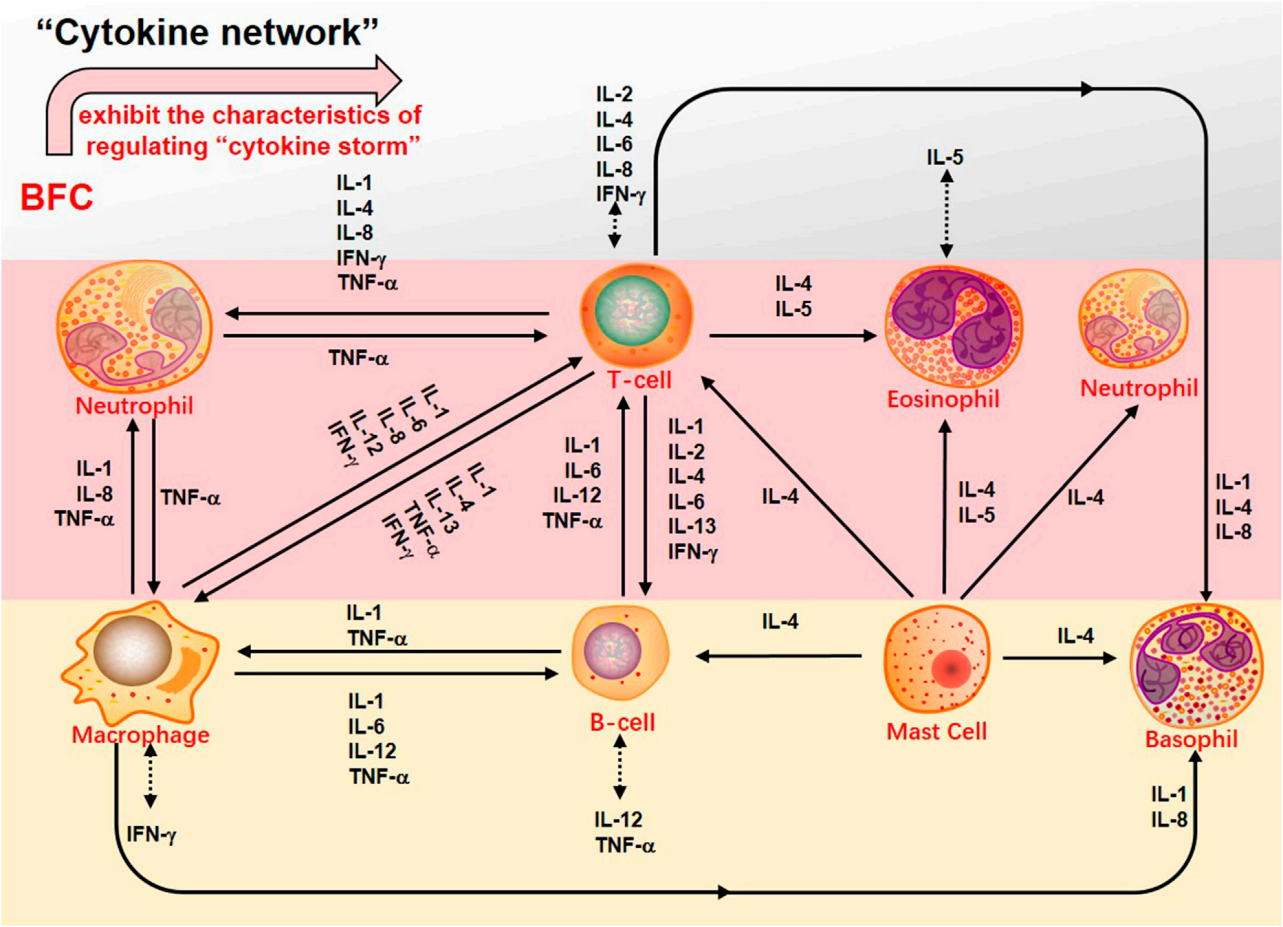
BFC exhibits the characteristics of regulating the “cytokine storm”.

Besides, the complications of serious COVID-19 involve severe pneumonia, pulmonary edema, ARDS, and organ failure (Wu and Mcgoogan, 2020). The pulmonary pathology primarily exerts distinct alveolar injury, such as alveolar edema and protein exudation meanwhile exhibits vascular congestion and inflammatory infiltration, along with local fibrin clusters mixed with mononuclear inflammatory cells and multinucleated giant cells (Tian et al., 2020). Thus, COVID-19 is usually associated with inflammation and characterized as acute inflammatory disease (Manjili et al., 2020). Recent reports indicated that severe COVID-19 patients had numerous evidently higher proinflammation cytokines levels of IL-6, TNF, IL-1, IL-2, IL-17, IFN-γ, G-CSF, MCP-1, IFN-γ-induced protein 10(IP-10), and so on, which was similar to that discovered in patients infected with SARS-CoV and MERS-CoV (Wong et al., 2004; Jiang et al., 2005; Lau et al., 2013; Wang et al., 2020c). Especially, severe forms of COVID-19 mainly refer to inflammatory cytokines IL-1β, TNF, and IL-6 (Fauter et al., 2020). Increased levels of inflammatory cytokines may influence lung function and elevated levels of specific cytokines in the pulmonary alveolar microenvironment may lead to respiratory distress. COVID-19 pneumonia is more prevailing in the elderly than in the younger (Yuki et al., 2020). With the increase of age, the ability to resist infection and protect immune responses reduces and the activity of macrophages declines. The level of pro-inflammatory cytokines increases, so that the severity of SARS-CoV-2 infection will worsen in the elderly (Linehan and Fitzgerald, 2015; Zhou et al., 2020b; De Virgiliis and Di Giovanni, 2020). Therefore, blocking these inflammatory cytokines can benefit patients with COVID-19. Victoriously suppressing inflammation may be a promising therapy for the treatment of COVID-19. What makes sense is that the preliminary try to ameliorate COVID-19 by blocking IL-6 reveals hope (Yang et al., 2020a; Liu et al., 2020d; Wang et al., 2020d). Pacha (Pacha et al., 2020) put forward that IL-17 was also a plausible target to decrease the recruitment of neutrophil. Through targeting on IL-17, some factors in ARDS would be suppressed. Studies found that the numbers of neutrophils and macrophages in the airways and blood of COVID-19 patients were increased (Wu et al., 2020a; Du et al., 2020) and the proliferation of macrophages was usually found. Meanwhile, COVID-19 patients showed infiltration of monocytes, lymphocytes, and neutrophils. Bridgewood (Bridgewood et al., 2020) suggested that severe inflammation in COVID-19 could be treated by blocking infiltration of neutrophils, monocytes, and lymphocytes, and decreasing inflammatory cytokines such as TNF-α, IL-6, IL-1β, and chemokines such as IFN, CCL-2 produced by immune cells, and airway epithelial cells. The alkaloids from BFC such as imperialine, verticinone, verticine, peimisine, and delavine could inhibit IL-1β, TNF-α, and IL-6 production, and suppress inflammatory response in the lung, which might help to alleviate ARDS associated with COVID-19 and fibrosis development. The characteristics of regulating the “cytokine storm” that BFC exhibits is shown (Figure 9).

COVID-19 cases have different types such as mild, moderate, severe, and critical cases (Saied et al., 2021). However, there are many COVID-19 patients who tested positive on the RT-PCR test for SARS-CoV-2 but with no typical clinical symptoms or signs (Wu et al., 2020e; Schuetz et al., 2020; Torres et al., 2021). These infections are asymptomatic but they can also transmit the virus to others (Luo et al., 2020a; Huang et al., 2020b). Studies have shown that the viral load that was detected in the asymptomatic patient was similar to that in the symptomatic patients and asymptomatic or mild cases combined represent about 40–50% of all infections (Qiu, 2020; Zou et al., 2020). It is a great importance to prevent and control this specific type of asymptomatic infection. Some asymptomatic infections may develop into symptomatic cases, and some will recover by themselves (Gao et al., 2021). Young cases (<15 years old) of COVID-19 such as children had milder clinical manifestations and nearly half of them were prone to be asymptomatic (Hu et al., 2020; Qiu et al., 2020). The pathological mechanism shows that adults have a much higher prevalence of increased C-reactive protein than do children, and there is less immune damage in children with a much milder immunological response (Qiu et al., 2020). Thus, in asymptomatic patients, SARS-CoV-2 invasion causes only a specific mild immune response. Besides, some researchers supported that antiviral therapy could fasten viral clearance on asymptomatic infections (Hu et al., 2020; Lu et al., 2021). BFC is natural drug sources for respiratory diseases from Fritillaria, and Fritillaria displays antiviral activities such as influenza viruses that cause respiratory diseases (Wang et al., 2021). Therefore, in nucleic acid screening for SARS-CoV-2 positive, diagnosed as asymptomatic infection, it is necessary to intervene by giving BFC for treatment the first time. Age and body condition may play an important role in the severity of COVID-19, which is related to different immune responses and other potential factors. BFC may have a benefit for COVID-19 patients with treatment of typical clinical symptoms such as fever, cough, sore throat, dyspnea, etc., exerting antiviral activities, and regulating of immune responses, inflammatory responses, STAT, NF-κB, and MAPK signaling pathways, the “cytokine storm”, and RAS (Table 2).

**TABLE 2:** The clinical characteristics and corresponding pathological or potential treatment mechanisms of COVID-19 intervened by BFC

NO.	Type	Nucleic acid tests	Clinical characteristics	Pathological/treatment mechanisms	Population trend	BFC intervention
1	Asymptomatic	positive	No clinical symptoms	• Immune responses	Young	*Yes*
			Others	• Antiviral therapy		
				• Others		
2	Mild	positive	Mild clinical symptoms	• Clinical symptom treatment		
			Others	• Lung inflammation		
				• Immune responses		
3	Moderate	positive	Clinical symptoms	• Inflammatory responses	Adult or elderly	
			Mild pneumonia	• Cytokine storm		
			Others	• Renin-angiotensin system (RAS), ACE2/ACE target		
4	Severe	positive	Clinical features	• STAT, NF-κB, and MAPK signaling pathways			
			Hypoxia	• Others		
			Rapid breath			
			Vital organs injuries			
			Others			
5	Critical	positive	Respiratory failure	• Mechanical ventilation		
			ARDS	• ICU monitoring treatment		
			Shock	• Medication		
			Other organ failure	• Others		
			Others			

## Summary and Outlook

**FIGURE 10:**
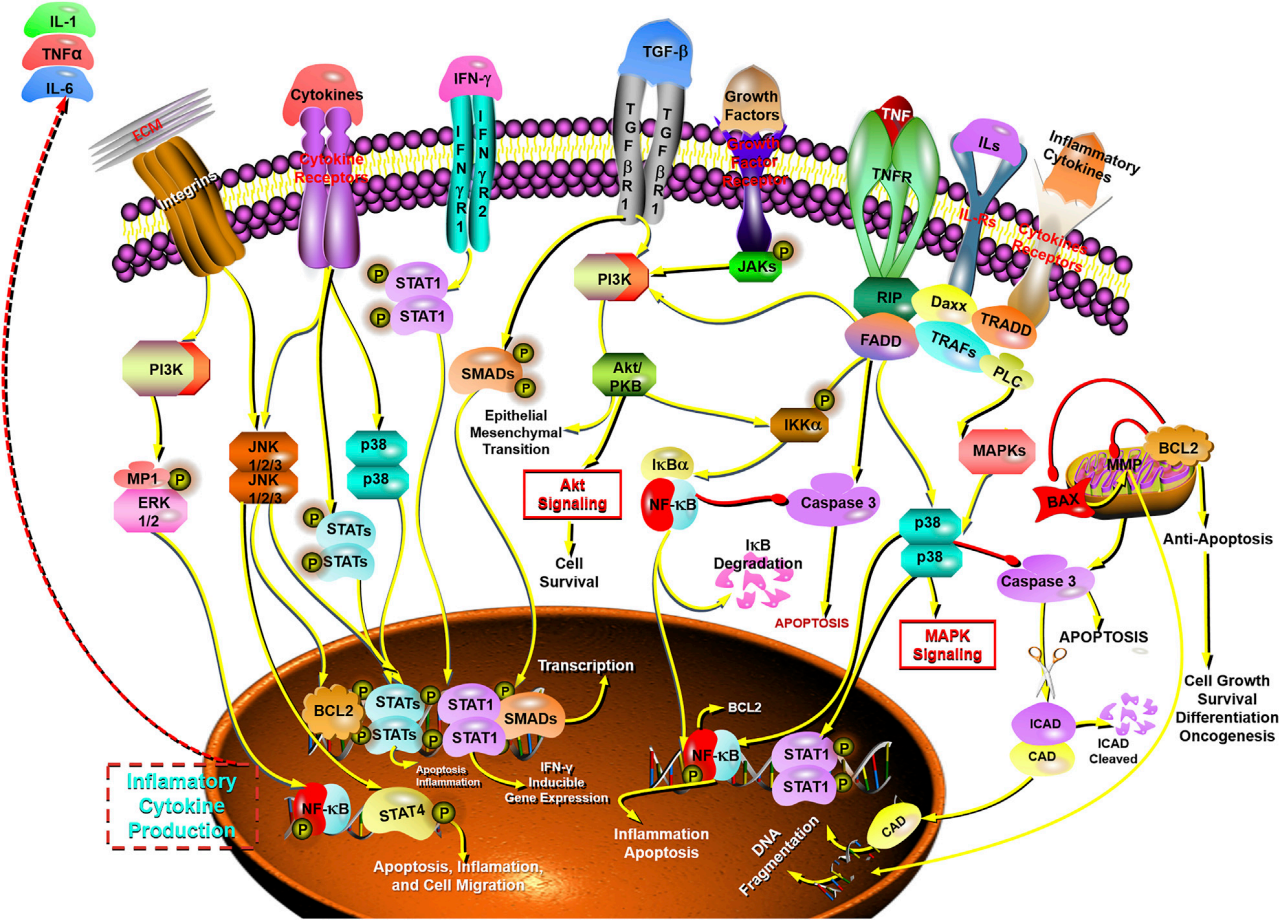
The main related signaling pathways of BFC’s effect on respiratory diseases.

In order to successfully defeat the life-threatening COVID-19, many efforts should be made to target ACE, block the cytokines storm, inhibit inflammation, modulate immunity, improve the symptoms, alleviate lung damage, and prevent pulmonary fibrosis. However, up to now, there is no strong clinical evidence to support the efficacy of any other drugs against COVID-19. Namely, there is still lack of specific drugs as well as therapeutic regimens for the treatment of COVID-19 except for the existing chemical drugs such as intravenous remdesivir and dexamethasone, which have modest effects and can only alleviate some symptoms (Zumla et al., 2016; Bailly and Vergoten, 2020; Li and De Clercq, 2020; Asselah et al., 2021). Nowadays, natural products are attracting more and more attention because they are the source of drugs for the prevention and treatment of many diseases. TCM is considered to be an essential source for discovering natural products with biological activity exerting minimal side effects and showing good results in many difficult problems. Through long-term research on different TCM, numerous valuable compounds have been discovered, such as artemisinin (Klayman, 1985; Tu, 2016), paclitaxel (Rowinsky and Donehower, 1995; Ma and Hidalgo, 2013), curcumin (Heger et al., 2013; Mirzaei et al., 2016; Shafabakhsh et al., 2019), berberine (Kong et al., 2004; Ortiz et al., 2014; Kong et al., 2020), phloretin (Aliomrani et al., 2016; Ye et al., 2018), and so on. Besides, TCM has achieved good clinical effects in the prevention and treatment of SARS-CoV, MERS-CoV, H1N1, H7N9, Ebola, and other epidemics (Liu et al., 2004; Li and Peng, 2013; Luo et al., 2019b; Lu et al., 2020b; Huang et al., 2021). It was worth noting that in 2003, TCM could shorten the hospitalization, reduce drug side effects, and improve symptoms of patients with SARS (World Health Organization, 2004; Leung, 2007). Furthermore, the genomic and in silico structural characterization of SARS-CoV-2 showed that SARS-CoV-2 and SARS-CoV were closely interrelated. It reminds us that TCM may have potential application value in the current COVID-19 epidemic, and it is a resource for drug discovery against SARS-CoV-2 (Ling, 2020). Currently, in the SARS-CoV-2 pandemic, TCM has played a big role in China’s fight against COVID-19 and in China more than 70,000 patients, or 92% of all confirmed cases on the mainland have received TCM treatment, which has been effective for over 90% of them (Zhang, 2020). In addition to conventional remedy, the intervention of TCM as a complementary therapy has also made a difference. Various medicines applied in TCM system have been recommended for the treatment of COVID-19, and the TCM therapy is on account of the different period of disease and symptoms (National Health Commission of the People’s Republic of China; Yang et al., 2020b). TCM is characterized by multiple components, multi-targets, multiple pathways, and exerts poly-pharmacological synergistic effect on the human body (Wang et al., 2012b; Guo et al., 2020b). The secondary plant metabolites in herbal TCM play a comprehensive therapeutic role and have participated in dealing with many complex diseases (Hussein and El Anssary, 2019). As the result of analogous chemical structure of secondary metabolites, there is a synergistic or similar pharmacological effect probably and compounds with similar activities can act on the same targets such as protein family in a synergistic manner against the redundancy of the biological network. Besides, compounds without common targets can also produce similar therapeutic effects on the same disease because different targets could be involved in the same signaling pathway, which is closely related to the pathological process (Ye et al., 2012). Therefore, it is of great significance to find potential Chinese medicines of anti-COVID-19 based on the chemical database of TCM (Luo et al., 2020b; Zhang et al., 2020c; Zhang and Liu, 2020). Even, it is believed, that the combination of TCM and Western medicine might be a potent therapeutic approach for COVID-19 (Zhang et al., 2004; Liu et al., 2020e). BFC, as an important edible and medicinal plant, has strong bioactive material basis. Modern pharmacological research shows that the extracts, alkaloid, or monomer alkaloids of BFC have extraordinary anti-inflammatory properties, which are helpful for the treatment of inflammation of the respiratory system. For example, BFC alkaloids imperialine, chuanbeinone, verticine, verticinone, and peimisine are used to treat lung-related diseases such as lung inflammation, COPD, ARDS, tuberculosis, and lung cancer. BFC has also been developed into herbal dietary supplement products such as the most popular Nin Jiom Pei Pa Koa used to relieve sore throat, cough, etc., which is mainly Fritillaria-based herbal extracts of sucrose syrup (Cunningham et al., 2018; Drugs.com, 1064b). Through this review, it is found that the therapeutic mechanism of BFC on respiratory diseases exerts the characteristics of multi-components, multi-targets, and multi-signaling pathways. The main related signaling pathways of BFC’s effect on respiratory diseases are shown (Figure 10). The pharmacological activities of BFC with details are summarized and shown in Table 3. The main related compounds and targets of BFC effect on respiratory diseases are shown as follows (Tables 4, 5). SARS-CoV-2 will cause inflammatory responses characterized by damage of deep airway and alveolar; meanwhile, the patients develop symptoms such as fever, dry cough, and fatigue, and the lungs eventually show fibrosis and exudative lesions. BFC has a good therapeutic effect on respiratory diseases to treat cough, sputum, asthma, bronchial inflammation, ARDS, COPD, and pneumonia due to its beneficial antitussive, expectorant, anti-inflammatory, anti-asthma, anti-oxidant, and anti-pneumonic effects. Then, we speculate that by utilizing the alkaloids in BFC to act on lung injury, lung inflammation, ACE target, immunoregulation, oxidative stress, and so on, is there a potential drug in the treatment of COVID-19? The reasons BFC may become a potential drug and be beneficial for COVID-19 are as follows:

① The diseases of the elderly and the patients with basic diseases, such as the immunocompromised are often serious and dangerous after being infected, and the mortality rate of the elderly over 65 years old is distinctly higher (Zheng et al., 2020b), while BFC is considered to be effective for the treatment of the elderly and children.
② The clinical manifestations of COVID-19 are fever, dry cough, phlegm, shortness of breath, etc., while BFC can manage the symptoms such as fever, dry cough, phlegm, asthma, and sore throat. Even as for the asymptomatic infections, it is necessary to intervene by giving BFC to treat the first time due to its antiviral activities, and regulation of immune responses in the early stage.
③ SARS-CoV-2 virus infection mainly affects the lungs, and the infected patients begin with flu-like symptoms, quickly developing to ARDS, while BFC exhibits significantly therapeutic effects on ARDS.
④ Lung inflammation is one of the characteristics of COVID-19, while BFC has evidently anti-inflammatory activity and obviously alleviates lung inflammation.
⑤ In the “cytokine storm” of SARS-CoV-2 infection, there is an intense inflammatory response with the release of abundant increased pro-inflammatory cytokines such as IL-1β, IL-7, IL-8, IL-9, IL-6, IL-10, FGF, G-CSF, GM-CSF, IFN-γ, IP-10, MCP-1, MIP-1A, MIP1-B, PDGF, TNF-α, and VEGF in COVID-19 patients, while BFC can inhibit IL-1β, IL-8, IFN-γ, TNF-α, IL-6, IL-5, IL-13, IL-4, NO, PGE2, COX-2, and iNOS production to suppress inflammatory response.
⑥ There is a serious immune imbalance in COVID-19 patients and the immune response is dysregulated and abnormally activated, while BFC has an effect on immunomodulation with regulating the immune related pathways, involving STAT, NF-κB, and MAPK signaling pathways.
⑦ ACE is involved in the disease process of COVID-19 and ACE inhibitors are considered to be a promising drug to treat COVID-19, while the alkaloids in BFC can inhibit the activity of ACE in a dose-dependent manner.

**TABLE 3:** The pharmacological activities of BFC with details.

Pharmacological effects	Components	Models	Details	Positive/negative control	Dose	Application	Ref.
Anti-tussive	Extraction with 80% ethanol solvent	KM mice were exposed to 25% NH_4_OH produced by a sprayer	Enhanced the latent period of cough and inhibited the cough frequency of mice	Dextromethorphan, 15 mg/kg	0.098–1.475 g/kg	*In vivo*	Xu et al. (2019)
	Imperialine, chuanbeinone, verticinone, verticine	Classical mouse cough model induced by ammonia liquor	Inhibited cough frequency and increased latent period of cough in mice induced by ammonia	Codeine phosphate, 30 mg/kg	1.5–3.0 mg/kg	*In vivo*	Wang et al. (2011)
	Imperialine, imperialine-β-N-oxide, isoverticine, isoverticine-β-N-oxide	Mouse cough model induced by ammonia liquor	Inhibited cough frequency and increased latent period of cough in mice induced by ammonia	Codeine phosphate, 30 mg/kg	1.5–4.5 mg/kg	*In vivo*	Wang et al. (2012a)
	Verticine	Computational target fishing combined with manual database mining	MAPK1, AKT1 and PPKCB were found for the important targets of cough	—	—	*In silico*	Zhang et al. (2020a)
**Expectorant**	Extraction with 80% ethanol solvent	Expectorant effects were evaluated by phenol red expectoration experiment with intraperitoneal injection of phenol red solution to KM mice	Enhanced tracheal phenol red output	Ambroxol hydrochloride, 15 mg/kg	0.098–1.475 g/kg	*In vivo*	Xu et al. (2019)
	Imperialine, verticinone, verticine	KM mice were treated with intraperitoneal injection of phenol red solution	Enhanced mice’s tracheal phenol red output	Ammonium chloride, 1500 mg/kg	1.5–3.0 mg/kg	*In vivo*	Wang et al. (2011)
	Imperialine, imperialine-β-N-oxide, isoverticine, isoverticine-β-N-oxide	KM mice were treated with intraperitoneal injection of phenol red solution	Enhanced mice’s tracheal phenol red output	Ammonium chloride, 1500 mg/kg	1.5–4.5 mg/kg	*In vivo*	Wang et al. (2012b)
**Anti-inflammatory**	Verticinone	Rat model of bleomycin-induced lung inflammation	Ameliorated inflammation of alveolar and lung interstitial, suppressed pulmonary fibrosis, down-regulated IFN-γ levels in serum and TGF-β, NF-κB, CTGF, ERK1/2, and FasL	Dexamethasone 0.000405 g/kg	0.005 g/kg	*In vivo*	Guo et al. (2013)
	Verticine	NCI-H292 cells induced by EGF, PMA, or TNF-α	Inhibited the expression of MUC5AC mucin gene and protein	—	10^−6^-10^−5^ M	*In vitro*	Kim et al. (2016)
	Extraction with 80% ethanol solvent	Xylene was applied to the anterior and posterior surfaces of mice ear	Inhibited ear edema induced by the xylene	Dexamethasone, 10 mg/kg	0.098–1.475 g/kg	*In vivo*	Xu et al. (2019)
	Imperialine, chuanbeinone	Ear edema model was induced by the xylene	Inhibited the development of ear edema	Dexamethasone, 5.25 mg/kg	1.5–3.0 mg/kg	*In vivo*	Wang et al. (2011)
	Imperialine, imperialine-β-N-oxide, isoverticine, isoverticine-β-N-oxide	Ear edema model was induced by the xylene	Inhibited the development of ear edema	Dexamethasone, 5.25–10.50 mg/kg	1.5–4.5 mg/kg	*In vivo*	Wang et al. (2012b)
	Total alkaloid fraction	Models of acetic acid-induced capillary permeability accentuation, cotton pellet-induced granuloma formation	Inhibited acetic acid-induced capillary permeability accentuation, cotton pellet-induced granuloma formation	Dexamethasone, 5 mg/kg	4.5–18 mg/kg	*In vivo*	Wang et al. (2016)
	Verticinone, imperialine	LPS-stimulated RAW 264.7 macrophages	Inhibited the production of NO, TNF-α, IL-1β and the expressions of iNOs and COX-2, decreased NF-κB	Berberine, 10 µM	100–600 µM	*In vitro*	Wu et al. (2015)
	Imperialine, verticinone, verticine, peimisine, delavine	LPS-induced RAW264.7 macrophage cells	Lowered the production of NO, TNF-α and IL-6, and inhibited the mRNA expressions of TNF-α and IL-6. Inhibited the phosphorylated activation of MAPK signaling pathways, included ERK1/2, p38 MAPK and JNK/SAPK	—	5–100 μM	*In vitro*	Liu et al. (2020a)
	Verticine	PMACI-induced human mast cell (HMC-1). SD rats were injected anti-DNP IgE and DNP-HAS	Attenuated the production of pro-inflammatory cytokines IL-6, IL-8, and TNF-α and reduced the phosphorylation of MAPKs and the expression of NF-κB, decreased PCA reactions	1–100 μg/ml Dexamethasone, 1 mg/kg	1–100 μg/ml, 1–5 mg/kg	*In vitro* and *in vivo*	Park et al. (2017)
	Verticine	Isolated HEK 293 cells were voltage clamped using a PC 505B patch clamp amplifier in the whole-cell configuration	Inhibited Kv1.3 ion channel and blocked the Nav1.7 ion channel	—	1–300 μM	*In vitro*	Xu et al. (2016)
	Verticinone	Murine models of inflammatory and neuropathic pain induced by acetic acid and rat model of paclitaxel induced neuropathic inflammatory pain	Inhibited acetic acid-induced writhing response, and the nociceptive response	200 mg/kg aspirin	1.5–3 mg/kg	*In vivo*	Xu et al. (2011)
	Verticine	IL-1β induced inflammatory response in mouse articular chondrocytes and ameliorates murine osteoarthritis model	Inhibited the expression of NO, PGE2, COX-2, TNF-α, iNOS, and IL-6. Increased the expression of aggrecan and collagen-II, alleviate the degradation of ECM and ADAMTS-5 and MMP-13. Inhibited AKT phosphorylation and NF-κB activation with the activation of Nrf2/HO-1 signaling pathways	—	10–50 μg/ml, 5 mg/kg	*In vitro* and *in vivo*	Luo et al. (2019a)
	Verticinone	LPS-induced mastitis model in mice and the mouse mammary epithelial cells (mMECs) model stimulated with LPS	Lowered the MPO activity, decreased the production of pro-inflammatory mediator TNF-α, IL-6, IL-1β, COX-2, and iNOS. Suppressed phosphorylation of AKT/NF-κB, ERK1/2, and p38 signaling pathways	—	1–5 mg/kg, 30–70 μg/ml	*In vivo* and *in vitro*	Gong et al. (2018)
**Anti-oxidant**	Verticinone, verticine, imperialine, imperialine-3-β-D-glucoside, delavine, peimisine	Cigarette smoke extract (CSE)-induced oxidative stress model in RAW264.7 cells	Down-regulated the production of ROS, up-regulated the level of GSH and expression of HO-1 and Nrf2	—	5–50 μM	*In vitro*	Liu et al. (2020)
**Anti-pulmonary fibrosis**	Verticinone	Rat model of bleomycin-induced pulmonary fibrosis	Ameliorated inflammation of alveolar and lung interstitial, suppressed pulmonary fibrosis, down-regulated IFN-γ levels in serum and TGF-β, NF-κB, CTGF, ERK1/2, and FasL	Dexamethasone, 0.000405 g/kg	0.005 g/kg	*In vivo*	Guo et al. (2013)
**Anti-cancer**	Verticinone	GBM cells model and BALB/c nude mice model was established by the injection of U251 cells into the right hips of each mouse	Inhibited glioblastoma via arresting the cell cycle and blocked autophagic flux, downregulated *p*-Akt and *p*-GSK3β, p-AMPK and *p*-ULK1	—	100–400 μM,2 mg/kg	*In vitro* and *in vivo*	Zhao et al. (2018)
	Verticinone	Colorectal cancer HCT-116 cell	Inhibited colorectal cancer cell proliferation by inducing apoptosis and autophagy and modulating key metabolic pathways	—	100–400 μM	*in vitro*	Zheng et al. (2017)
	Verticinone	Human promyelocytic leukemia HL-60 cells	Inhibited the cell growth by inducing these cells to differentiate toward granulocytes	All-trans retinoic acid (ATRA), 0.1–1.0 μM	1–10 μM	*In vitro*	Pae et al. (2002)
	Verticinone	Immortalized keratinocytes and oral cancer cells	Inhibited growth, induced apoptosis and G0G1 cell cycle arrest, down-regulated Bcl-2 and up-regulated Bax, activated caspase-3 through a caspase pathway mediated by mitochondrial damage	—	1–50 μg/ml	*In vitro*	Yun et al. (2008)
	Water extraction	Ovarian and endometrial cancer cells	Decreased cell growth on soft agar and decreased the invasive potential of cancer cells. Activated caspase-3, G0/G1 phase cell cycle arrest, and downregulated cyclins D1 and D3 and induction of p27. Decreased NF-κB DNA binding, reduced expression of p- IκBa, abrogated NF-κB activation, and downregulated NF-κB-regulated metastasis-promoting proteins	—	200 μg/ml	*In vitro*	Kavandi et al. (2015)
	Verticinone, verticine	KM mice model bearing a 7-day tumor with injection subcutaneously in the right axilla of mice for the solid form	Inhibited the growth of the solid type of hepatoma in mice	5-fluorouracil, 10 mg/kg	1.25–5 mg/kg	*In vivo*	Li et al. (1995)
	Water extraction	Human endometrial cancer cell lines Ishikawa and HEC-1B	Decreased in expression of TGF-β isoforms, TGF-β receptors, and SMADs. Inhibited basal and TGF-β1-induced cancer cell proliferation and invasion, with abrogation of snail, slug, MMPs, αvβ3 integrin, FAK, and *p*-FAK expression	—	200 μg/ml	*In vitro*	Bokhari and Syed (2015)
**Anti-asthma**	BFC extracted by water	C57BL/6 mice model of asthma	Down-regulated the levels of IL-5, IL-13, and IL-4 in the bronchoalveolar lavage fluid and reduced the level of ovalbumin-specific IgE in serum. Lowered the number of eosinophils by inhibiting the recruitment of eosinophil and airway inflammation	Cyclosporin A, 20 mg/kg	200 mg/kg	*In vivo*	Yeum et al., 2007
	Imperaline, sinpeinine A, 3β-acetylimperialine	CHO-hM2 and CHO-hM3 cells	Acted as the antagonists of selective muscarinic M2 receptor subtype and selective muscarinic M3 receptor subtype antagonist	—	1–10 μM	*In vitro*	Lin et al. (2006)
**Anti-COPD**	Imperialine	COPD-like rat model induced by the combination of exposure to cigarette smoke (CS) and intratracheal administration of LPS	Alleviated the injury of lung function and structure to reduce the progression of COPD. Inhibited inflammatory response in the lung by regulating the expression of IL-1β, IL-6, IL-8, TNF-α, NF-kB, TGF-β1, MMP-9, and TIMP-1	Dexamethasone sodium phosphate (DSP), 1.0 mg/kg	3.5–7.0 mg/kg	*In vivo*	Wang et al. (2016)
**Anti-ARDS**	Total alkaloid fraction of BFC	LPS-induced ARDS in C57 mice	Suppressed inflammatory cells recruitment and cytokine (TNF and IL-6) production in the bronchoalveolar lavage fluid, and attenuated pathological changes in the lung tissues of ARDS mice	DEX, 5 mg/kg	15–60 mg/kg	*In vivo*	Wang et al. (2016)
**Anti-lung cancer**	Total alkaloids of BFC, peimisine, imperialine, chuanbeinone	Human lung carcinoma cell line (A549)	Antiproliferative effect, suppressed tumor angiogenesis and promoted apoptosis through increasing the level of caspase-3 expression	—	40–80 μg/ml	*In vitro*	Wang et al. (2014)
	Imperialine	A549 tumor-bearing BALB/c nude mouse model	Suppressed both NSCLC tumor and associated inflammation through an inflammation-cancer feedback loop, inhibited NF-κB activity	—	10 mg/kg	*In vivo*	Lin et al. (2020)
	Aqueous extract	NSCLC A549 cells model and xenograft model of nude mice	Inhibited A549 cells proliferation and colony formation and increased the expressions of STAT 1 and STAT4 and their target genes IFN-γ and IL-12, triggered Bcl-2/Bax proteins attributing to cellular apoptosis, lessened the size of the tumor and induced cytokines IL-12 and IFN-γ secretion	—	100 μg/ml 2.5 mg/ml	*In vitro* and *in vivo*	Li et al. (2020b)
	Total alkaloids of BFC, peimisine, imperialine, chuanbeinone	Lewis lung carcinoma cells (LLC) model, the mice models were constructed by inoculating LLC cells suspension into the left armpit of C57BL/6 J mice	Inhibited tumor angiogenesis and induced apoptosis through activating caspase-3	Cyclophosphamide, 20 mg/kg	4–64 μg/ml 10–40 mg/kg	*In vitro* and *in vivo*	Wang et al. (2014)
	Chuanbeinone	Lewis lung carcinoma cells (LLC) model and LLC cells were subcutaneously inoculated into the left armpit of the mice model	Induced S phase arrest and apoptosis, decreased the antiapoptotic Bcl-2 expression and increased the proapoptotic protein Bax and caspase-3 expression to suppress tumor angiogenesis	Mitoxantrone hydrochloride, 5 μg/ml cyclophosphamide, 20 mg/kg	5–15 μg/ml,10–40 mg/kg	*In vitro* and *in vivo*	Wang et al. (2015)
**Anti-pulmonary tuberculosis**	Feitai Capsule containing BFC	Patients with pulmonary tuberculosis	Promoted the absorption of the lesions and the closure of cavities, and accelerated the conversion of sputum bacteria to negative, enhanced the immunity, and promoted the inflammation dissipation of patients	—	2.5 g each time, 3 times per day	*In vivo*	Wang et al. (2017)
	Niubeixiaohe containing BFC	Tuberculosis mice models infected with Mycobacterium tuberculosis H37Rv	Improved the pulmonary lesions to return to normal pulmonary structure	22.5 μg *M. vaccae* vaccine	1.5–26.6 mg/ml	*In vivo*	Liang et al. (2017)

**TABLE 4:** The main related compounds of BFC’s effect on respiratory diseases

Components	No.	Compounds	2D structure	No.	Compounds	2D structure	No.	Compounds	2D structure
Alkaloids	1	Verticinone	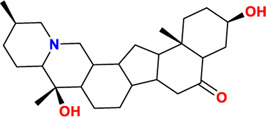	2	Imperialine	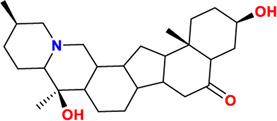	3	Isoverticine-β-N-oxide	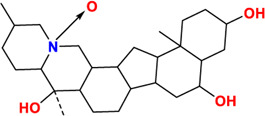
	4	Verticine	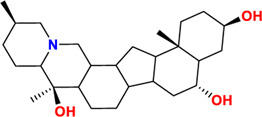	5	Chuanbeinone	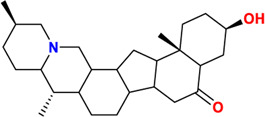	6	Imperialine-β-N-oxide	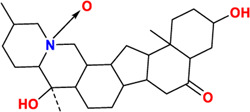
	7	Isoverticine	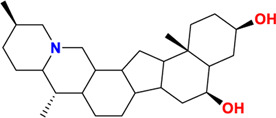	8	Peimisine	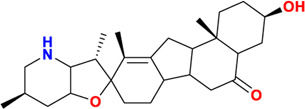	9	Imperialine-3-β-D-glucoside	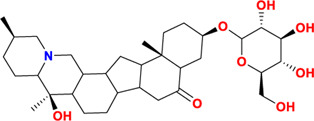
	10	Delavine	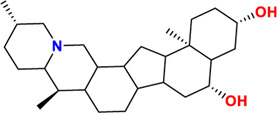	11	Sinpeinine A	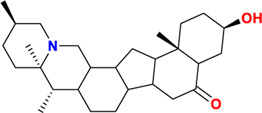			

**TABLE 5:** The main related targets of BFC effect on respiratory diseases

No.	Targets (PDB ID)	3D structure	No.	Targets (PDB ID)	3D structure	No.	Targets (PDB ID)	3D structure	No.	Targets (PDB ID)	3D structure
1	IL-1β (2WRY)	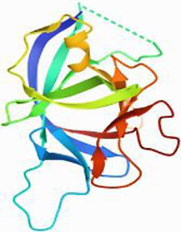	2	TNF-α (5MU8)	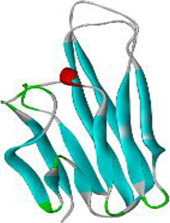	3	IFN-γ (6F1E)	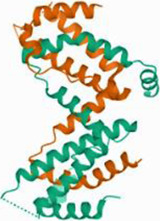	4	STAT1 (1YVL)	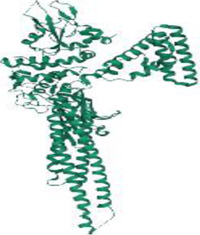
5	IL-4 (2B8U)	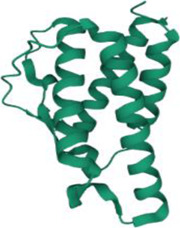	6	STAT4 (1BGF)	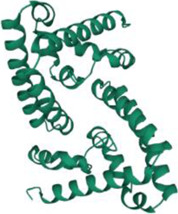	7	AKT (6S9W)	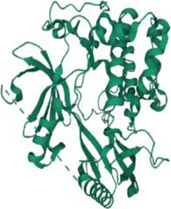	8	iNOS (1DD7)	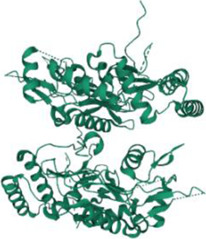
9	IL-5 (1HUL)	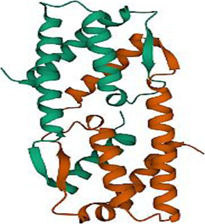	10	MMP-9 (2OW1)	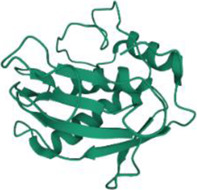	11	MAPK (3UIB)	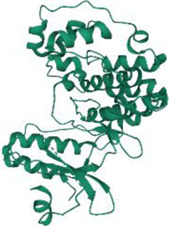	12	TGF-β1 (1KLD)	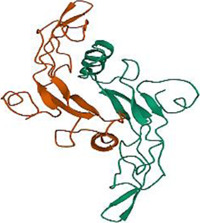
13	IL-6 (7NXZ)	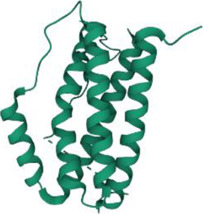	14	MMP-13 (1FLS)	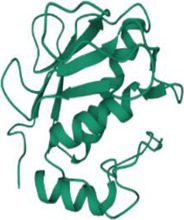	15	HO-1 (1NI6)	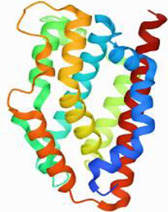	16	Caspase-3 (1RHJ)	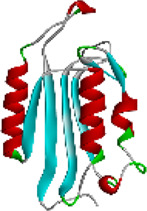
17	IL-8 (1ICW)	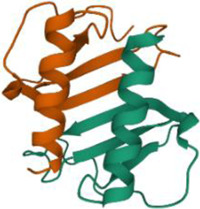	18	TIMP-1 (1UEA)	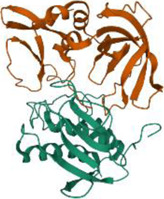	19	Nrf2 (7K2L)	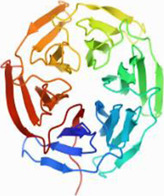	20	Bcl-2 (5JSN)	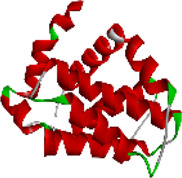
21	MUC5AC mucin gene (5AJO)	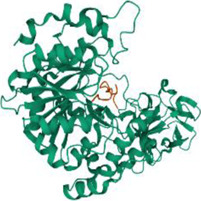	22	PGE2 (2PBJ)	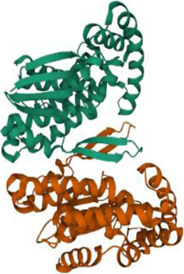	23	COX-2 (5FDQ)	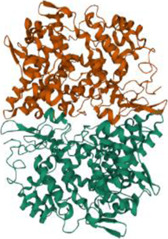	24	Bax (4BD6)	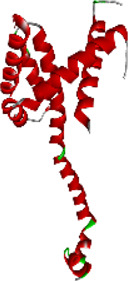
25	IL-12 (1F42)	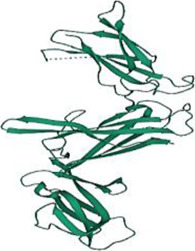	26	p38 (1WFC)	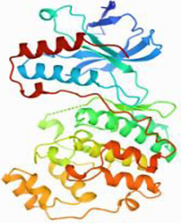	27	ERK1/2 (6G9A)	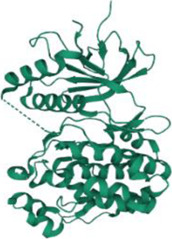	28	NF-κB (p65/P50) (1LE5)	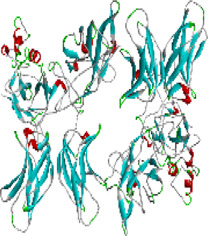
29	IL-13 (1IJZ)	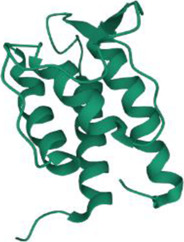	30	FasL (5L19)	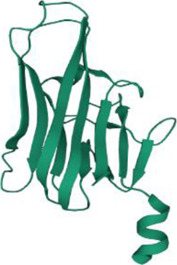	31	MDR (3G5U)	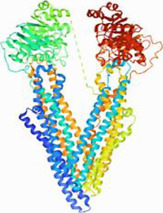	32	Aggrecan (1TDQ)	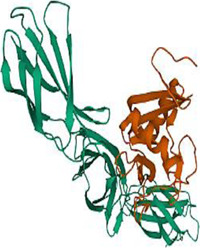
33	ACE (1O86)	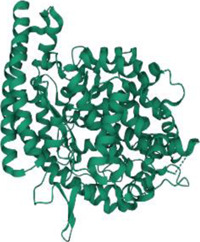	34	MPO (7LAG)	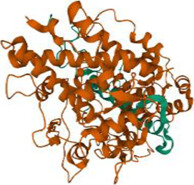	35	ERCC1 (2A1I)	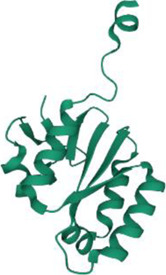	36	Kv1.3 ion channel (4BGC)	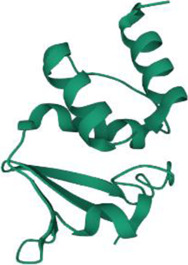
37	JNK/SAPK (4UX9)	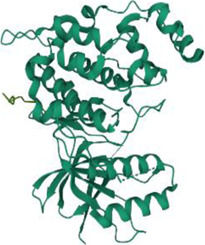	38	IgE (1FP5)	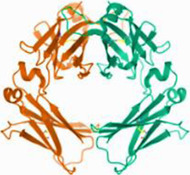	39	ADAMTS-5 (3B8Z)	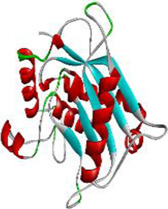	40	Nav1.7 ion channel (6J8G)	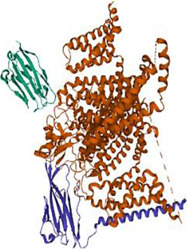
41	Collagen-II (6HG7)	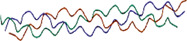	42	muscarinic M2 receptor (5ZK3)	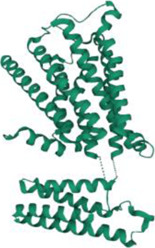	43	Muscarinic M3 receptor (4DAJ)	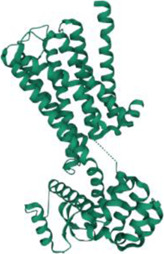	44	Collagen-II (5NIR)	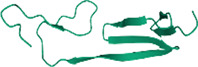
45	GSH	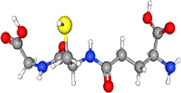	46	histamine	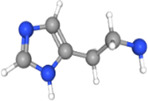	47	ROS	O2·-, H2O2, ·OH	48	PCA reactions	—
49	LRP	—	50	CTGF	—	51	PPKCB	—	52	ECM	—
53	NO	—									

In summary, BFC has unique advantages in the prevention and treatment of respiratory diseases, which is worthy of in-depth excavation and application. For COVID-19, may it be a potential drug? This review expounds the potentiality theoretically, but it still needs to be verified by a large number of experiments. COVID-19 has become a major threat to the health of people all over the world, we hope this review can provide insights on the drug discovery of anti-COVID-19.
